# Region-selective and site-specific glycation of influenza proteins surrounding the viral envelope membrane

**DOI:** 10.1038/s41598-024-69793-7

**Published:** 2024-08-16

**Authors:** Yi-Min She, Zongchao Jia, Xu Zhang

**Affiliations:** 1 Centre for Oncology, Radiopharmaceuticals and Research, Biologic and Radiopharmaceutical Drugs Directorate, Health Canada, Ottawa, ON K1A 0K9 Canada; 2 Department of Biomedical and Molecular Sciences, Queen’s University, Kingston, ON K7L 3N6 Canada; 3 School of Pharmaceutical Sciences, Faculty of Medicine, University of Ottawa, Ottawa, ON K1H 8M5 Canada

**Keywords:** Mass spectrometry, Glycosylation

## Abstract

Analysis of protein modifications is critical for quality control of therapeutic biologics. However, the identification and quantification of naturally occurring glycation of membrane proteins by mass spectrometry remain technically challenging. We used highly sensitive LC MS/MS analyses combined with multiple enzyme digestions to determine low abundance early-stage lysine glycation products of influenza vaccines derived from embryonated chicken eggs and cultured cells. Straightforward sequencing was enhanced by MS/MS fragmentation of small peptides. As a result, we determined a widespread distribution of lysine modifications attributed by the region-selectivity and site-specificity of glycation toward influenza matrix 1, hemagglutinin and neuraminidase. Topological analysis provides insights into the site-specific lysine glycation, localizing in the distinct structural regions of proteins surrounding the viral envelope membrane. Our finding highlights the proteome-wide discovery of lysine glycation of influenza membrane proteins and potential effects on the structural assembly, stability, receptor binding and enzyme activity, demonstrating that the impacts of accumulated glycation on the quality of products can be directly monitored by mass spectrometry-based structural proteomics analyses.

## Introduction

Glycation, involving non-enzymatic reactions of reducing sugars or sugar-derived metabolites with the amine group of protein N-terminus, lysine and arginine residues, is a common post-translational modification of proteins in human metabolism and therapeutic drugs^1,2^. The early-stage glycation adducts are reversible, which can undergo Maillard reactions, Amadori and Heyns rearrangement to form stable advanced glycation end products (AGEs)^3^. AGEs have direct impacts on a large number of biological processes including metabolic dysfunction, chronic diseases, aging and age-related disorders^1,4–6^. Glycation is typically caused by chemical reactions of proteins in cell culture media and endogenous metabolites of protein expression^2^, and storage exposure to reducing sugars^7^, resulting in undesired modifications and negative effects on the quality, efficiency and safety of therapeutic biologics. For example, site-specific glycation of recombinant proteins in monoclonal antibody drugs affects protein stability, degradation, aggregation, antigen binding, half-life and immunogenicity^2,8^, and is therefore considered as a potential critical quality attribute that must be monitored and controlled. Despite its biological importance, the impairment of glycation on the protein structure and function of influenza vaccine products has hitherto not been explored. Vaccination is an effective method of preventing virus infections, with the most common influenza vaccines primarily produced by live attenuated or inactivated viruses growing in embryonated chicken eggs or cultured cells^9^. In the manufacturing processes, quality control of protein modifications resulting from in vivo biosynthesis and in vitro chemical reactions is essential for ensuring the safety and efficacy of vaccine products. The presence of high abundance product-related *N*-glycosylation of glycoproteins in influenza vaccines and process-induced modifications by β-propiolactone (BPL) and formaldehyde during virus inactivation has been extensively investigated by us and other research groups^10–14^.

Efforts to achieve in-depth characterization of protein modifications of influenza vaccines, focusing on the previously uncharacterized glycation, are currently undertaken to evaluate the potential impacts on the quality of products. Liquid chromatography coupled to tandem mass spectrometry (LC MS/MS) is utilized for the highly efficient separation and sensitive detection of glycated peptides following enzymatic digestions of proteins. Nevertheless, large-scale analysis of naturally occurring glycation products of influenza membrane proteins remains a technical challenge due to the lability of modifications, low abundances, poor peptide fragmentation and insufficient sequencing coverage^15^. Poor MS/MS fragmentation often occurs for large-size glycated peptides containing missed trypsin cleavage sites, owing to a hexose group covalently attached to the lysine residue^16^. Ambiguous identity may be thus retrieved by automatic database search and peptide sequencing of incomplete MS/MS fragments in the case of *O*-linked glucosylation and mannosylation incurred at nearby serine/threonine residues, as the isobaric mass shift of 162 Da in a glycosylated residue is the same as that of lysine hexose-glycation (i.e. hexosylation)^17,18^. To overcome these problems, alternative techniques of boronate-affinity enrichment and electron transfer dissociation (ETD) were previously employed to improve the reliable identification of in vitro glucose-induced glycation sites of proteins^19–22^, however, the wide application was restricted by small amounts of material, low levels of native glycation and the relatively low ETD fragmentation efficiency of the glycated peptide backbone compared to that of collision-induced dissociation (CID). Although the use of ETD enables the reliable identification of large peptides containing labile post-translational modifications at multiply charged states, the peptide fragmentation by transferring electrons to protonated peptides is less sensitive than CID and ineffective for doubly charged ions and small peptides.

Herein, we present a straightforward method for the comprehensive characterization of early-stage protein glycation in influenza vaccines. High abundance *N*-glycan attachments of influenza glycoproteins were first removed by endoglycosidase PNGase F, and multiple enzyme digestions of proteins were then conducted at neutral pH conditions to generate small sized peptides for enhancing MS/MS fragmentations. Validation of the glycation sites were confirmed by manual inspection of MS/MS fragmentations of the glycated peptides based on sufficient sequencing information. Through examination of several vaccine strains of influenza A (H1N1, H3N2, H5N1, H7N9) and influenza B derived from mammalian Madin-Darby Canine Kidney (MDCK) cells, human embryonic retinal cells (Per C6) and embryonated chicken eggs, we have identified a widespread distribution of early-stage hexose-glycated lysines exclusively presented in matrix 1 (M1), hemagglutinin (HA) and neuraminidase (NA) surrounding the influenza envelop membrane. Structure-based topology analyses revealed that the site-specific glycation residues are highly conserved in the protein sequences across various subtypes of influenza vaccine strains. These residues are predominantly situated within distinct α-helix, β-sheet and loop regions, which are accessible to reducing sugar molecules. The critical sites of lysine glycation, localized at the transmembrane surface of protein M1 and in the receptor-binding cavity of HA and NA, are postulated to have significant effects on the structure and function of the proteins including stability, receptor binding and enzymatic activity.

## Results and discussion

### Site-specific lysine glycation of influenza proteins

Twelve influenza vaccines derived from embryonated chicken eggs and cultured cells were chosen for analyses to examine the possible glycation of proteins. The influenza virion typically contains proteins assembled by hemagglutinin (HA), neuraminidase (NA), matrix protein M1, ion channel membrane protein M2, non-structural proteins (NS1 and NS2), nucleoprotein (NP), ribonucleoprotein (RNP), nuclear export protein (NEP), polymerase acidic protein (PA), polymerase basic proteins (PB1 and PB2)^23,24^. Preliminary LC MS/MS analysis of protein digests, resulted from the sequential de-*N*-glycosylation by endoglycosidase PNGase F followed by cleavage using a single protease of trypsin or chymotrypsin, only determined partial sequences of the membrane proteins. Instead, combined enzymatic digestions with trypsin, endoproteinase Glu-C and chymotrypsin at neutral pH conditions were able to yield a nearly complete sequence coverage of individual proteins. Initial screening of protein modifications was performed using Byonic wide search against the influenza proteome databases and glycan libraries, which retrieved a number of peptides bearing additional masses of 162 Da. In-depth investigation of the modified peptides by Mascot search identified the glycation at lysine residues. These glycated peptides were remarkably detected in the proteins to varying degrees in eight vaccine samples (Tables S1, S2). By comparison, high levels of glycated proteins were observed in influenza A/Brisbane/10/2010 (H1N1), A/Hong Kong/4801/2014 (H3N2), A/turkey/Turkey/1/2005 (H5N1), B/Brisbane/60/2008 (egg-derived) and B/Phuket/3073/2013, whereas the lysine glycation of influenza A/Michigan/45/2015 (H1N1), A/Anhui/1/2013 (H7N9) and B/Brisbane/60/2008 (Per. C6 cell-derived) appeared at lower frequencies. Exceptionally, influenza A/Michigan/45/2015 has only one glycation site occupied at Lys187 of M1. It is of note that the glycation of peptides merely occurred at the specific lysine sites of the membrane proteins M1, HA and NA, rather than the other structural proteins of M2, NP, RNP, NS1, NS2, NEP, PA, PB1 and PB2, suggesting the region-selectivity and site-specificity of influenza protein glycation surrounding the viral envelop membrane.

**Figure 1:**
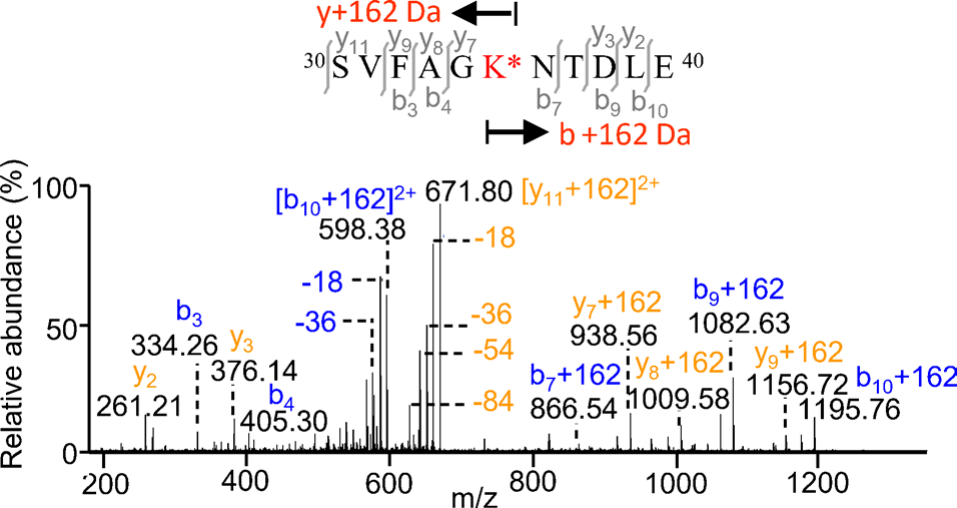
MS/MS spectrum of a glycated peptide from an endoproteinase Glu-C/trypsin digest of influenza A/Brisbane/10/2010. Peptide sequencing of the doubly charged ions of m/z 671.8223 identified the 162 Da modification at Lys35 in the peptide 30–40 of matrix 1. The mass spectrum is analyzed using Thermo Xcalibur 4.1.50.

To obtain the reliable identification of glycated peptides and the precise location of glycation sites, we conducted a thorough inspection of the database search results by means of high mass-accuracy measurements, MS/MS fragmentation patterns, and the existence of missed trypsin cleavage sites of peptides due to lysine glycation^25^. In agree with previous reports^26,27^, MS/MS fragmentation of glycated peptides displayed characteristic fragments involving the neutral losses of water molecules (− 18 Da, − 36 Da, − 54 Da) from the peptide precursor ions, owing to preferential cleavages of the attached hexose moiety to a glycated lysine under CID conditions. Figure 1 showed a typical MS/MS spectrum of the doubly charged ions of peptide 30–40 (SVFAG**K**^35^NTDLE) at m/z 671.8223, the presence of a series of singly charged b and y ions, along with the aforementioned neutral losses, identified the hexose glycation site at Lys35 (+ 162 Da) of the M1 peptide from an endoproteinase Glu-C and trypsin digest of influenza A/Brisbane/10/2010. Therefore, the use of multiple enzyme digestions and LC MS/MS sequencing of small peptides renders comprehensive identification of lysine glycation sites in proteins. Through such analyses, we have identified 23 unique lysine glycation sites of M1 (9), HA (10) and NA (4) in the influenza A/Brisbane/10/2010 (Supplementary Fig. S1 and Table S1). Our observation based on manual data analysis of the MS/MS spectra has shown no serine/threonine O-glycation in the peptides, consistent with the previous report^28^.

### Preferential glycation sites of the inner membrane protein M1 at Lys35 and Lys187

**Figure 2:**
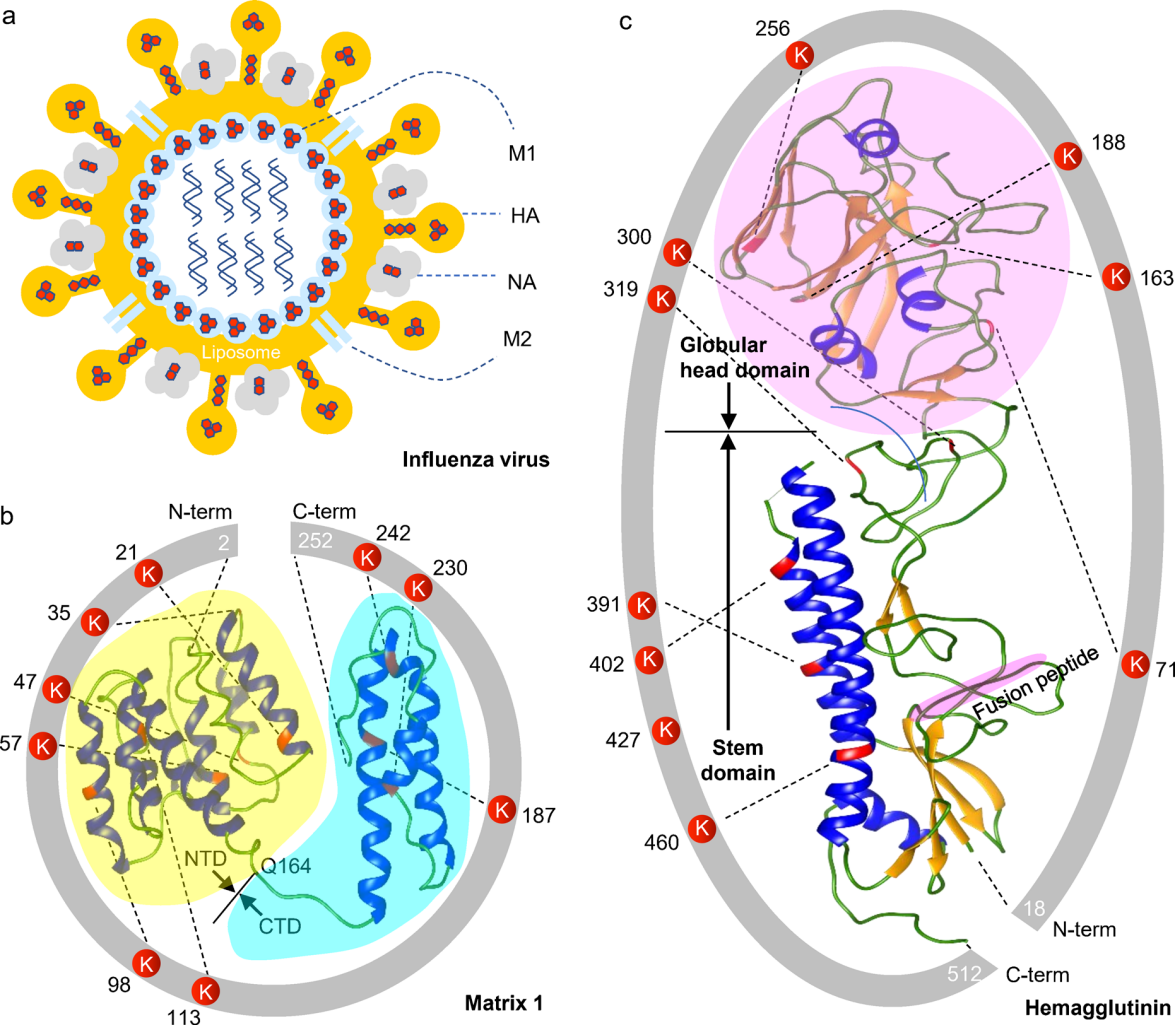
Topological localization of glycated proteins in influenza A/Brisbane/10/2010. (**a**) The typical influenza virus contains several proteins including two major surface glycoproteins hemagglutinin (HA) and neuraminidase (NA), the matrix 1 (M1) inside the virus envelope, and the ion channel membrane protein (M2) across the membrane. A hexagon shape diagram in the inset represents multi-hexose glycation of lysines in the membrane proteins of M1, HA and NA. (**b**) The structure of M1 is composed of 252 amino acids with the deletion of methionine at the N-terminus, consisting of an N-terminal domain (NTD, residues 2–163) and a C-terminal domain (CTD, residues 164–252). (**c**) The HA structure encompasses the globular head domain (i.e. the receptor binding domain, RBD) and the stem domain. The glycated lysines at the structural locations are highlighted in red. The protein structure modeling and visualization are made using Phyre2 server (http://www.sbg.bio.ic.ac.uk/phyre2/) and UCSF Chimera 1.15 (https://www.cgl.ucsf.edu/chimera/), respectively.

Influenza protein M1 is the most abundant structural protein located inside the viral lipid bilayer membrane, and plays a vital role in budding and stabilizing virus particles (Fig. 2a)^29,30^. Sequences of M1 are highly conserved in the influenza virus family (Fig. S2), and the structure typically contains a globular N-terminal domain (NTD, residues 1–164) and a flexible C-terminal domain (CTD, residues 165–252)^31–33^. Positively charged residues on the surface of the NTD electrostatically interact with negatively charged lipid molecules of the viral envelope membrane, revealing a high affinity of liposome binding^34,35^. In influenza A/Brisbane/10/2010, the full-length sequence of M1 consists of 13 lysines (Fig. S2), in which 9 of them were found to be glycated at residues Lys21, Lys57, Lys98, Lys113, Lys187, Lys230 and Lys242 in the helix bundles and Lys35, Lys47 in the loop regions (Fig. 2b).

**Figure 3:**
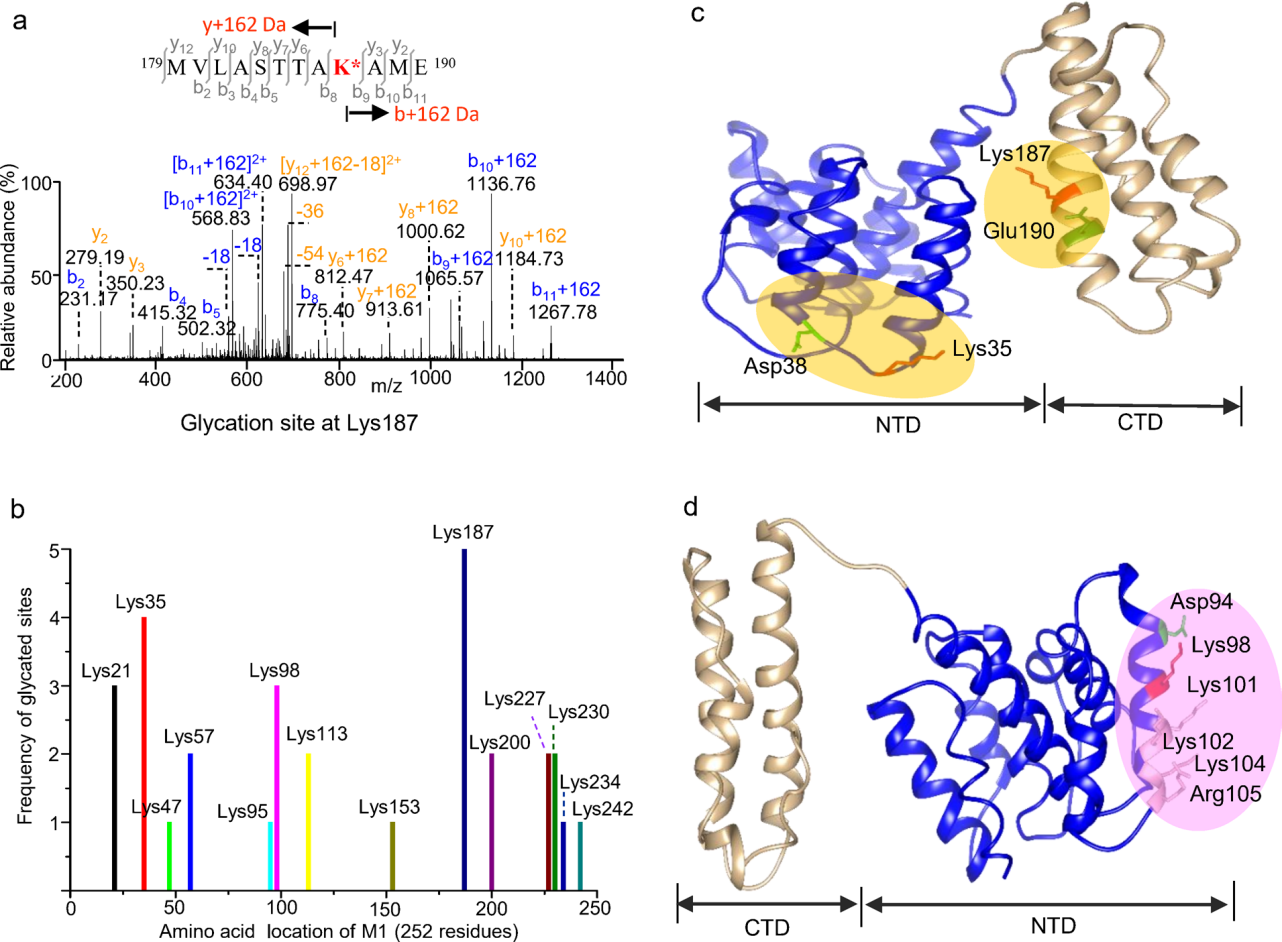
Neighboring group effects on the glycation of Lys35, Lys98 and Lys187 in matrix 1. (**a**) MS/MS sequencing of the doubly charged ion of m/z 707.8440, from an endoproteinase Glu-C/trypsin digest of influenza A/Brisbane/10/2010 (H1N1), identified the glycation site of peptide 179–190 at Lys187. (**b**) Distribution of M1 glycated residues in influenza vaccines. (**c**) The proximate acidic residue Glu190 branching in parallel toward Lys187 on the same helical face catalyzes Lys187 glycation, while the spatial location of adjacent Asp38 branching on the flanked loop in an antiparallel orientation toward Lys35 is favorable for Lys35 glycation. (**d**) The neighboring Asp94 catalyzes Lys98 glycation. However, the highly electrostatic interaction of multiple basic residues with the membrane lipid bilayer prohibits glycation of lysines 101, 102 and 104 nearby Lys98. The domain organization of M1: 2–163, NTD; 164–252, CTD. The mass spectrum is analyzed using Thermo Xcalibur 4.1.50, and the protein structure is visualized by UCSF Chimera 1.15 (https://www.cgl.ucsf.edu/chimera/).

The preferential glycation sites of M1 are localized at the highly conserved residues Lys35 and Lys187 (Fig. S2), as determined to be the higher and highest occurring frequencies of glycated residues in the influenza vaccines analyzed (Fig. 3a,b). Early-stage glycation is catalyzed by the neighboring group interaction of acidic residues in the vicinity of lysine residues^36,37^, and is favored by stabilizing helical conformations in which the proximate position of catalytic groups is spatially aligned on the same helix face as that of lysine^38,39^. The sequences of the glycated peptides contain the structural motifs of ^35^KXXD^38^, ^94^DXXXK^98^ and ^187^KXXE^190^, in which the neighboring Glu190 to Lys187 branched in parallel on the helical membrane surface that catalyzes the Lys187 glycation, whereas the adjacent Asp38 to Lys35 extended its branching side chain flanked on the loop region in an antiparallel orientation and catalyzed the Lys35 glycation (Fig. 3c). Similarly, the neighboring Asp94 catalyzes the Lys98 glycation on the helical surface (Fig. 3d). The existence of nonglycated lysines at residues 101, 102 and 104, adjacent to Lys98, could be ascribed to the spatial location in the region without catalytic residues. The glycation could be also inhibited by tightly electrostatic interactions of the surface charges between multiple basic residues (Lys101, Lys102, Lys104 and Arg105) and lipid molecules in the viral membrane (Fig. 3d). Overall, the wide-spread lysine glycation of M1 can lead to the extensive removal of positive surface charges of the α-helical bundles, which has the potential to disrupt the interactions with the lipid membrane as well as the associated viral proteins, and consequently destabilize the structural integrity of influenza virus assembly.

### Structural impacts of lysine glycation on outer membrane proteins hemagglutinin and neuraminidase

Influenza HA and NA are two major surface glycoproteins. HA is responsible for attachment of the virus to cell surface receptors and subsequent fusion between the viral envelope and the host cell membrane, while NA cleaves sialic acids (SA) from glycoproteins enabling the virus particles to be released from the cells^40^. Cleavage of HA precursor (HA0) into two subunits (HA1 and HA2) in the mature protein by an endogenous protease is essential, which occurs at a site characterized by the presence of basic amino acids and conserved residues^41,42^. HA1 initializes the receptor binding at the early stage of virus infection and HA2 mediates cell membrane fusion^43^. Structural assembling of HA homotrimer comprises a highly variable globular head domain (GHD) and a conserved stem domain in each monomer^44,45^. The GHD contains a receptor-binding pocket, responsible for binding SA receptors, and 4 antigenic sites (Sa, Sb, Ca and Cb) that are recognized by the host immune system. In the HA structure of influenza A/Brisbane/10/2010 (Fig. 2c), the involvement of 4 loops at residues 132–165, 168–178, 183–189, 252–255 in the GHD contains 11 lysines with positive charges enables the electrostatic interactions with SA receptors. Of the identified 10 glycation sites, Lys71, Lys163 and Lys188 are situated within the flanked loop regions of the GHD, and Lys256 is positioned at the transition from a β-strand to the short loop linker extension spanning residues 252–255. These glycated lysines localize around the receptor-binding cavity, and thus could possess potential effects on the receptor binding of HA. In view of the antigenic sites reported previously (Fig. S3)^45^, the glycated lysine residues are not distributed in the targeted antigenic sequence regions and consequently may unveil no functional impairment on the epitopes exposed for antibody recognition (Fig. S4).

In addition to receptor binding, HA also functions in mediating membrane fusion in virus infection. In this role, the stem domain folds in a central triple-stranded coiled-coil structure followed by a loop region and two antiparallel helices that anchor HA to the viral membrane. The glycation of lysines 391, 402 and 460, localized on two major stalk membrane helices, could trigger the conformational change of the protein required for membrane fusion, although proteolytic cleavage sites at the highly conserved region involving a basic residue Arg344 in the vicinity of hydrophobic fusion peptide ^345^GLFGAIAGFIEGGW^358^ remain unaffected (Fig. 2c, Fig. S5).

**Figure 4:**
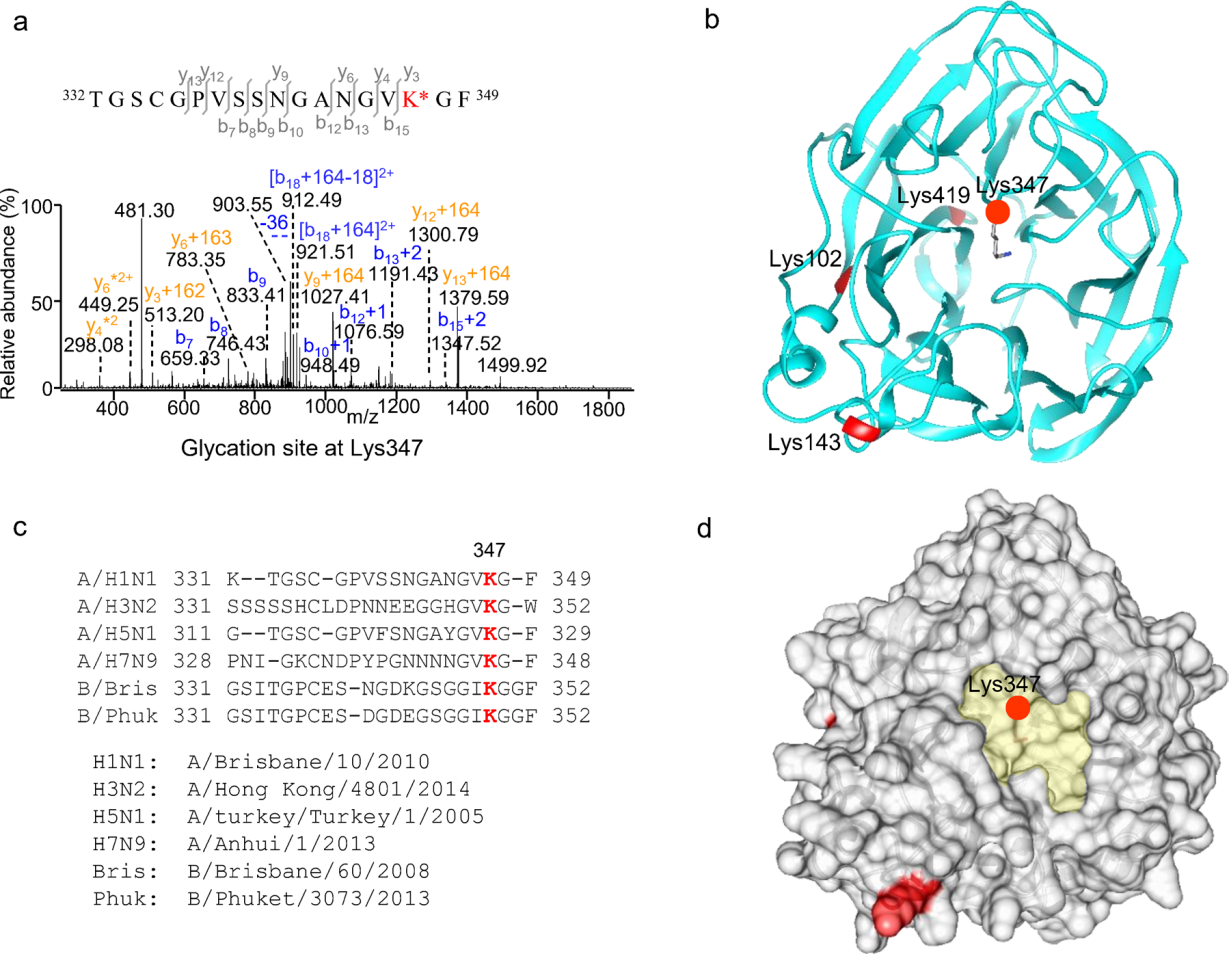
Lysine glycation sites of neuraminidase in influenza A/Brisbane/10/2010. (**a**) MS/MS sequencing of the doubly charged peptide ion at m/z 930.4031 from a chymotrypsin and trypsin digest identifies the hexose-glycation of peptide 332–349 at Lys347; (**b**) Multiple sequence alignment of neuraminidases of influenza NAs shows that the residue Lys347 is highly conserved; (**c**) The identified glycation sites at lysines 102, 143, 347 and 419 (coloured in red) by LC MS/MS are localized on the protein structure; (**d**) The surface topology shows the glycated Lys347 in the cavity and the receptor-binding pocket as highlighted with the atom stick model. The mass spectrum is analyzed using Thermo Xcalibur 4.1.50, and the protein structure is visualized by UCSF Chimera 1.15 (https://www.cgl.ucsf.edu/chimera/).

In contrast, the NA assembles as a homotetramer anchored to the viral envelope by the N-terminal hydrophobic transmembrane domain, consisting of GHD with the SA binding cavity surrounded by the 150 loop and 330 loop adjacent to the enzymatic active sites^46,47^. Four lysine glycation sites were found in the NA GHD of influenza A/Brisbane/10/2010 (Table S1, Fig. 4a), in which lysines 143, 102 and 419 are positioned at the α-helix and β-sheets of the protein surface, and the remaining Lys347 resides at the 330 loop region in the receptor binding cavity (Fig. 4b). The unmodified Lys347 naturally bearing a positive charge serves as a critical active site for SA binding, and the multiple sequence alignment of NAs indicated that Lys347 is highly conserved among influenza A and B virus strains (Fig. 4c,d and Fig. S6). As a result, Lys347 glycation could play a significant role to disrupt the interaction of NA with SA receptors and consequently influence the overall enzymatic activity. A comparable situation occurred at glycated Lys343 of NA in influenza B vaccines in the same structure region, as the reaction site is catalyzed by the neighboring residue in the protein (Table S2, Fig. 4c). Taken together, structural analyses suggested that the lysine glycation of the outer membrane proteins HA and NA may possess a detrimental effect on the receptor binding and enzyme activity. However, it does not seem to interfere with the antigenic sites recognized by antibody or the fusion peptide cleavage during the process of membrane fusion.

### Sequence-dependent lysine glycation of membrane proteins catalyzed by proximate residues

**Figure 5:**
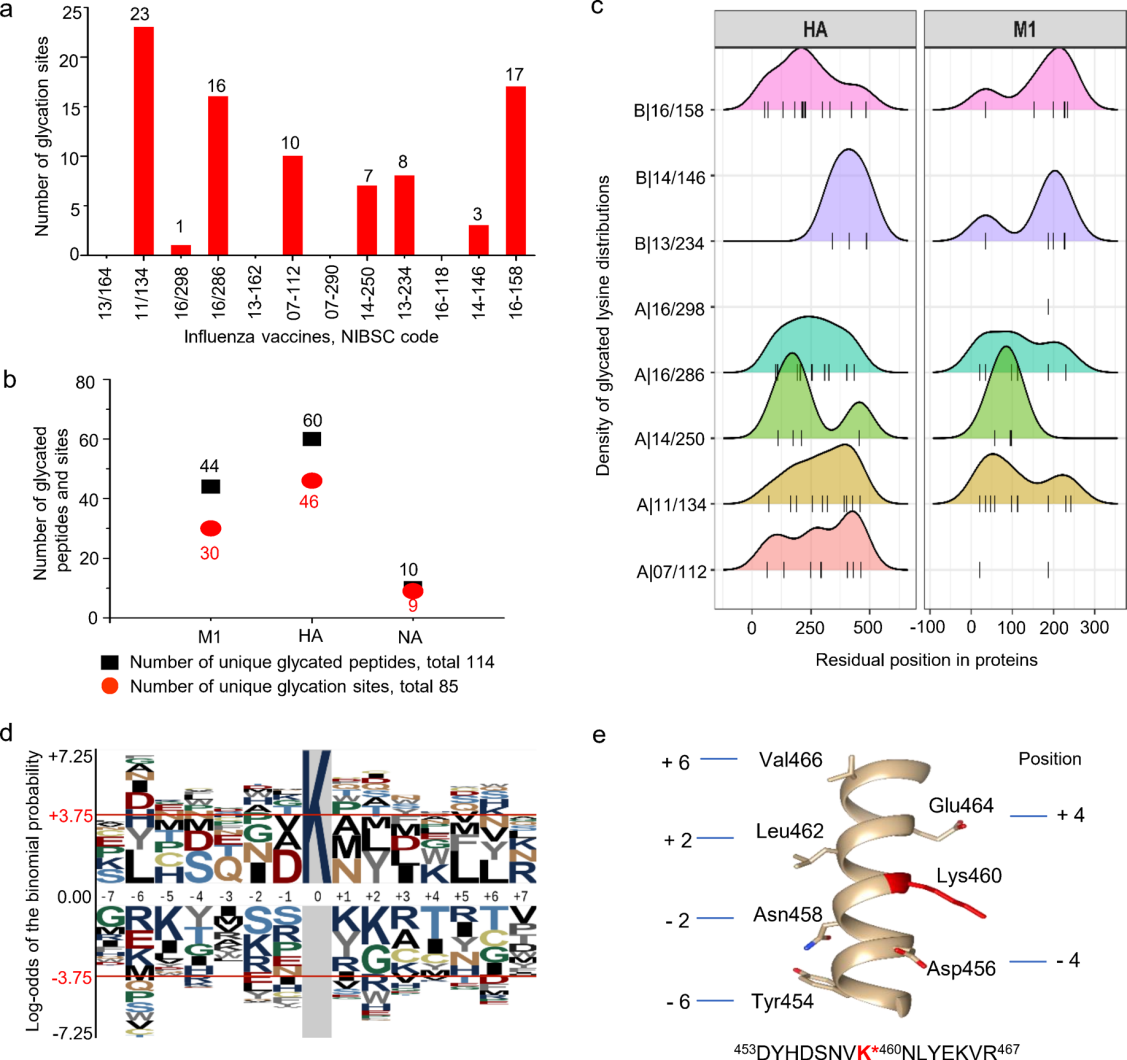
Sequence-dependent glycation sites of influenza vaccines. (**a**) The number of identified glycation sites in 12 influenza vaccine strains by LC MS/MS analyses; (**b**) The total number of glycated unique peptides and unique glycation sites of matrix 1 (M1), haemagglutinin (HA) and neuraminidase (NA); (**c**) The density of glycated lysine distributions in the protein sequences. Identified sites were indicated with vertical line (|) at the bottom of each sample panel. (**d**) The output of sequence logos generated by pLogo algorithm (https://plogo.uconn.edu/) from the 85 glycated sites of M1, HA and NA using the full influenza sequences as the background (p value < 0.05). The upper panel on positive y-axis is shown the overrepresented residues, and the lower panel on the negative y-axis is shown the underrepresented residues. (**e**) Spatial position of Lys460 (the HA glycation site of influenza A/Brisbane/10/2010) and the catalytic aspartic acid and glutamic acid in the helix chain of 15 residues. The structure is visualized by UCSF Chimera 1.15 (https://www.cgl.ucsf.edu/chimera/).

Similar measurements were performed on other vaccine strains, large-scale LC MS/MS analysis identified 114 unique glycated peptides and 85 glycated lysine sites of the membrane proteins M1, HA and NA (Table S2, Fig. 5a,b). Overall, the results displayed a wide-spread distribution of glycation in the proteins, and the site density analysis showed a clearly different profile in the NTD and CTD regions of M1 between the subtypes of influenza A and B (Fig. 5c). To investigate whether these glycation sites exhibit a statistical correlation between sequence patterns and neighboring residues, we first analyzed the relative frequency of 15 amino acid residues at the N- and C-terminal side of the glycation sites using the full influenza protein sequences as the background. Sequence logos showed no consensus sequence motif of lysine glycation, and no significant feature of the alignment on a high frequency of stretched residues from the glycation sites of membrane proteins (Fig. 5d). Considering the spatial effects of proximate residues on the formation of a glycated lysine on the same side of α-helical peptide surface, we then manually examined the polar residues at the ± 4 positions away from the glycated sites (Table S3). In the refined 85 sequence fragments, 24 of them were found to contain Ser/Thr residues, and 15 and 16 sequences consist of Asp/Glu and Lys/Arg at the proximate positions, respectively. As protein folding tends to retain hydrophilic residues on the outside of a protein structure, positively charged basic residues (Lys/Arg) on the protein surface often maintain tightly electrostatic interactions with lipids of the membrane and inhibit lysine glycation. Consequently, the uncharged Ser/Thr containing the hydroxyl side chain and negatively charged Asp/Glu at the ± 4 positions function as hydrogen bonding and the hydrophilic interactions with reducing sugars, catalyzing the non-enzymatic glycation of lysines on the helical surface. On the opposite side of the helix, residues lie at ± 2 and ± 6 positions spatially far away from lysine as shown in Fig. 5e. The motif-based sequence analysis revealed the overrepresented pattern of nonpolar residues (Leu, Ile, Phe, Val, etc.) surrounding the lysine glycation sites at ± 2 and ± 6 positions (Fig. 5d), indicating the participation of hydrophobic interactions of neighboring residues to stabilize the α-helical conformation. Our analyses thus identified the catalytic effects of adjacent residues on site-specific lysines, and the glycation of membrane proteins prefers to lysine sites localized at a helical region structurally bearing hydrophobic interior and hydrophilic surface.

### Limitations in the identification of influenza protein glycation

In addition to lysine glycation, Mascot database searches retrieved several arginine modifications of peptides by hexose-glycation in influenza proteins. Figure S7 showed the MS/MS spectrum of the doubly charged ion at m/z 478.7920 from a chymotrypsin digest of human Per. C6 cell-derived influenza B/Brisbane/60/2008. MS/MS fragmentation revealed a set of b_3_ to b_7_ product ions, corresponding to the predicted peptide fragments at residues 353–359 (RPPAKLL) of HA with the additional mass increase of 162 Da. Based on two pairs of complementary fragments between y_2_/b_5_ and y_3_/b_4_, the unchanged residual mass at Lys357 indicated that the lysine was not glycated. The modification is therefore inferred to the hexosylation of Arg353 at the N-terminus, rather than its nearby low-reactivity amino acids 354–356 (PPA). The signature assignments on two doubly charged fragment ions of [b_7_ + 162 Da] at m/z 469.80 and [b_7_ + 162 Da − 54 Da] (i.e. the losses of three water molecules) at m/z 442.28, further supported the identity of a hexose attachment to the peptide. Although arginine is able to form AGEs by either cellular metabolites or the degraded intermediates of monosaccharide autoxidation, it is less likely to be glycated via the early-stage non-enzymatic reaction with glucose^48^; the incidence of 162 Da mass increment could be explained by the glycosylation of arginine in the human cells catalyzed by a glycosyltransferase. Obviously, the formation of hexose-modified arginine here is unusual, and the underlying mechanism remains to be further understood.

Following careful examination of MS/MS spectra, we also found that other modified peptides containing a mass difference of 163 Da were mistakenly assigned to two modifications involving a hexose-glycated arginine (+ 162.0528 Da) and the deamidation of asparagine or glutamine (+ 0.9840 Da) (Fig. S8). The false-positive identifications were caused by the predicted peptides from a chymotrypsin digest of proteins, in which a tyrosine residue (+ 163.0633 Da) at the termini were actually missed in the sequences (HA residues 193–206 and NA residues 324–349). The MS/MS spectra revealed the mass increase of 163 Da exclusively in the series of either C-terminal y fragment ions (Fig. S8a) or N-terminal b fragment ions (Fig. S8b), suggesting the addition of tyrosine at the C- or N-terminus of the two peptides. Conceivably, alternative peptide sequence assignments of ^193^GIHHPNDAAEQTRL**Y**^207^ of HA in influenza A/turkey/Turkey/1/2005 and ^323^**Y**LDTPRPNDGSITGPCESDGDEGSGGIK^349^ of NA in influenza B/Phuket/3073/2013 were actually true to reflect the high-accuracy mass measurements and MS/MS fragmentations. Such incorrect identification can be avoided in the database search using a peptide mass error tolerance of less than 5 ppm, similar to that reported previously by Ma et al.^49^.

### Limitations of the study and potential solutions

In this study, a LC MS/MS method has been established for the proteome-wide identification of lysine glycation in influenza vaccines, and we demonstrated that the potential impacts of accumulated glycation on the quality of products can be monitored by mass spectrometry-based structural proteomics. Influenza vaccines samples were randomly selected from those derived from embryonated chicken eggs and cultured cells, and unbiased identification of the protein glycation was achieved by LC MS/MS analyses of protein digests, database search and manual validation of mass spectra. Subsequently, topological analyses of the identified glycation sites were conducted based on structural modeling of proteins from the cryo-EM and 3D crystal structures of a H1N1 2009 pandemic influenza virus. However, the method currently has some limitations. First, relative quantification of protein glycation in influenza vaccines is not feasible due to low abundances of native glycation products and large variations of protein sequences among the vaccine strains. Second, database search and manual data analysis at the proteomic scale can be time-consuming if non-specific enzymatic cleavages of membrane proteins are further considered. Third, the involvement of other types of modifications on proteins may interfere with the identification of low abundance lysine glycation, careful interpretation of datasets is thus required due to isobaric masses of peptides and ambiguous locations of modified residues caused by incomplete mass spectral fragmentation for MS/MS sequencing. Future investigations would be possible to develop alternative approaches by incorporating isotopically labeled standards of proteins or glucose into samples for accurate quantification of protein glycation. In addition, the throughput of data analyses could be improved by the degree of increasing specificity of enzymatic digestions, and sequential cleavages of proteins using the stepwise addition of individual proteases at the optimum pH conditions, instead of an enzyme mixture, result in high digestion efficiency of proteins. To enhance the accuracy of peptide identifications, the utilization of high mass accuracy measurements (< 5 ppm) of peptide precursor ions can reduce uncertainties and false positives in the database search results as mentioned above. In the presence of modified proteins on multiple amino acids by diverse functional groups, a pretreatment step of samples by eliminating unrelated protein modifications may help mitigate the effect. The removal of other predominantly coexisting modifications of proteins is also favorable to boost the detection of low abundance lysine glycation by mass spectrometry, similar to that of protein de-N-glycosylation in the experimental procedure.

## Conclusions

The extent of naturally occurring glycation of membrane proteins in influenza vaccine products is typically low, presenting a difficult task for analysis. We have determined the labile early-stage lysine glycation and mapped the topological location of influenza membrane proteins M1, HA and NA using highly sensitive LC MS/MS techniques. The non-enzymatic reactions of the proteins with reducing sugars are likely originated from either glucose in culture cell media or glucose and fructose generated from the hydrolysis of sucrose in influenza vaccine formulations, as the large polar metabolites of monosaccharides in embryonated chicken eggs and cultured cells cannot penetrate through lipid membrane without carrier proteins (i.e. transporters)^50,51^. Glucose-induced glycation can be an important quality attribute for certain therapeutic proteins^2,52^. The region-selectivity and site-specificity of the lysine glycation of influenza proteins surrounding the viral lipid membrane have potential effects on the protein structure, stability, virus assembly, receptor binding and enzyme activity. Accumulation of AGEs on lysine, arginine and cysteine residues by dicarbonyl metabolites is not evaluated in this study. Data analyses were hampered by the complexity of protein modifications involving high abundance process-induced BPL derivatives caused by virus inactivation^10^, which contain exactly the same compositions and isobaric mass changes of mono- and bis-alkylated modifications (C_3_H_4_O_2_, 72.0211 Da; C_6_H_8_O_4_, 144.0423 Da) as those of AGE products (e.g. N^ε^-carboxyethyl lysine (CML), N^ε^-carboxyethyl cysteine (CMC), tetrahydropyrimidine (THP) derivatives of arginine). In addition, the identical masses caused by the carbamidomethylation of cysteine and lysine (57.0215 Da) and the deamidation of asparagine/glutamine (0.9840 Da) during sample preparations also interfere with the global analysis of AGEs on carboxymethyl lysine (CML), carboxymethyl arginine (CMA) and carboxymethyl cysteine (CMC) (C_2_H_2_O_2_, 58.0055 Da).

In summary, we identified a wide range of early-stage lysine glycation, predominantly occurring at the highly conserved sites of influenza membrane proteins, especially the critical residues of Lys187, Lys163, Lys347 in the surface transmembrane domains of M1 and the receptor-binding domains of HA and NA, respectively. These findings reveal potential functional significance of lysine glycation of influenza proteins, and highlight the importance of avoiding such undesired reactions to ensure the quality, safety and efficacy of influenza vaccine products. The motif-based sequence analysis provides insights into the preferential sites of lysine glycation catalyzed by site-specific neighboring residues, contributing a broader understanding of glycation in membrane proteins.

## Methods and materials

### Materials

Influenza vaccines, stored in a freezer at − 80 °C, were originally obtained from the National Institute for Biological Standards and Control (NIBSC, Hertfordshire, UK) (Table S4). Specifically, influenza antigens of A/Brisbane/10/2010 (H1N1-like, 11/134), A/Texas/50/2012 (NMYC X-223A, H3N2-like, 13/162), A/turkey/Turkey/1/2005 (NIBRG-23, H5N1, 07/112), A/Anhui/1/2013 (NIBRG-268, H7N9, 14/250), B/Brisbane/60/2008 (NYMC BX-35, 14/146) were derived from mammalian MDCK and human Per C6 cells, respectively. Influenza antigens of A /California/7/2009 (NYMC X-179A, H1N1, 13/164), A/Michigan/45/2015 (NYMC X-275, H1N1, 16/298), A/Hong Kong/4801/2014 (NYMC X-263B, H3N2, 16/286), B/Brisbane/60/2008 (16/118), B/Brisbane/60/2008 (13/234), B/Phuket/3073/2013 (16/158) were prepared from purified virus grown in eggs. All vaccines were inactivated by BPL or formalin, suspended in phosphate-buffered saline solution containing 1% (w/v) sucrose and processed for freeze drying as described in the webpage (https://nibsc.org/documents).

Chemical reagents including acetonitrile (ACN), ammonium bicarbonate (NH_4_HCO_3_), dithiothreitol (DTT), iodoacetamide, tris(2-carboxyethyl)phosphine (TCEP), *N*-ethylmaleimide (NEM), glutamic acid, formic acid (FA), trifluoroacetic acid (TFA) were purchased from Sigma-Aldrich (Oakville, ON). Enzymes of sequencing-grade trypsin (porcine pancreas), endoproteinase Glu-C (staphylococcus aureaus V8), chymotrypsin (bovine pancreas), PNGase F (Elizabethkingia miricola) were obtained from Promega (Madison, WI), Roche Diagnostics (GmbH, Germany) and New England Biolabs (Whitby, ON), respectively.

### Protein purification and enzymatic digestions at neutral pH conditions

Vaccine samples were initially dissolved in 300 µL of 25 mM NH_4_HCO_3_, and dialyzed against 5 mM NH_4_HCO_3_ to remove low-molecular-weight compounds. Protein disulfide bonds were reduced with 10 mM DTT at 60 °C and alkylated with 55 mM iodoacetamide for 1 h each. The samples were dialyzed again and dried by a refrigerated CentriVap concentrator (Labconco, Fort Scott, KS). Subsequently, the alkylated proteins in 25 mM NH_4_HCO_3_ were deglycosylated by addition of 1 uL of PNGase F and incubated at 37 °C overnight. Enzymatic digestion of proteins was performed at 37 °C overnight using trypsin, or endoproteinase Glu-C, chymotrypsin followed by the addition of trypsin at the enzyme-to-protein substrate ratio of 1/100 (w/w), 1/50 (w/w) and 1/50 (w/w) in 25 mM NH_4_HCO_3_ at pH 7.6 for another 12 h, respectively.

### LC MS/MS analysis of enzymatic digests

The enzymatic digests of influenza proteins were dried, dissolved in 0.2% FA, and analyzed by data dependent LC MS/MS on an Orbitrap Fusion (Thermo Fisher Scientific Inc., Watham, MA) coupled with an Acquity ultra-performance liquid chromatography M-class system (Waters, Milford, MA). Peptides were trapped for 3 min at a flow rate of 5 µL min^−1^ of solvent A (0.1% FA in water) on a NanoEase M/Z symmetry C18 trap column (180 µm × 2 cm, 5 µm, 100 Å), followed by separation with either a NanoEase M/Z HSS C18 T3 column (75 µm × 15 cm, 1.8 µm, 100 Å) or a peptide BEH C18 column (75 µm × 10 cm, 1.7 µm, 130 Å) at 250 nL min^−1^ using a linear gradient of 10–30% solvent B (0.1% FA in acetonitrile) over 65 min. MS survey scans were acquired by the Orbitrap at a resolution of 120,000 at the mass range of m/z 350 to 1800 at the positive ionization mode. MS/MS measurements were carried out on multiply charged ions of peptides using the ion-trap and helium gas at low-energy collision-induced dissociation (CID), normalized collision energies of 27%. Dynamic exclusion was enabled for a period of 30 s.

### Identification of glycated peptides of influenza proteins

Influenza proteome databases were created using protein sequences downloaded from the Global Initiative on Sharing All Influenza Data (GISAID) EpiFlu database (https://www.gisaid.org). Raw LC MS/MS data and the converted mascot generic format (mgf) files using Proteome Discoverer (Thermo Fisher Scientific Inc.) were used to search for glycated peptides by Byonic (version 3.6.0, Protein Metrics Inc.) and Mascot (version 2.7.0, Matrix Science) software. To simplify data interpretation, search parameters were set to the enzymatic cleavage specificity sites of trypsin at lysine and arginine, endoproteinase Glu-C at aspartic acid and glutamic acid, chymotrypsin at phenylalanine, tryptophan, tyrosine and leucine. The maximum missed cleavage of enzymes was set to 3. Deamidation of asparagine or glutamine (+ 0.9840 Da) and the glycation of lysine and arginine (+ 162.0528 Da) were considered for variable modifications. Carbamidomethylation (+ 57.0215 Da) of cysteine was set to the fixed modification. Mass tolerances were set to 10 ppm for Orbitrap MS ions, and 0.8 Da for CID ion-trap MS/MS fragments. The significance threshold of peptide identifications was chosen to be p < 0.05 for Mascot search. A false discovery rate (FDR) was set to 1% for Byonic in peptide assignments. The identified glycated peptides were carefully examined by Xcalibur software (version 4.1.50, Thermo Fisher Scientific Inc.) and validated by manual inspection of mass spectra based on the predicted MS/MS peptide fragments and the high mass accuracy (~ 1 ppm) of MS measurements.

### Topological analysis of lysine glycation of influenza proteins

Structural modeling of proteins in the influenza A/Brisbane/10/2010 was built using Phyre2 server (http://www.sbg.bio.ic.ac.uk/phyre2/) with 100% confidence, and confirmed using AlphaFold (https://alphafold.ebi.ac.uk). Templates of M1, HA and NA were adopted based on the cryo-EM and 3D crystal structures of H1N1 2009 pandemic influenza virus in the Protein Data Bank (PDB) with the accession numbers of 7JM3, 5K9O and 4B7Q, respectively^31,53,54^. The topological location of lysine glycation in the protein structures was analyzed using UCSF Chimera 1.15 (https://www.cgl.ucsf.edu/chimera/).

### Statistical analysis of glycated lysine sites

Sequence logos were generated by pLogo algorithm (https://plogo.uconn.edu/) using a sequence window of 15 amino acid residues surrounding glycation sites. Since the identified glycation sites were restricted to proteins M1, HA and NA, full sequences of the three influenza proteins were used as the background for statistical analysis. The glycation site distribution plot was generated using ggplot in R (version 4.2.2) and the density was calculated using R package ggridges.

### Supplementary Information

Supplementary Information.

## Data Availability

The mass spectrometry proteomics data support the findings of this study have been deposited to the ProteomeXchange Consortium via the PRIDE partner repository with the primary accession code PXD052096.
