# Supplementary material for: Region-selective and site-specific glycation of influenza proteins surrounding the viral envelope membrane
Source: Sci Rep. 2024 Aug 16;14:18975. doi: 10.1038/s41598-024-69793-7 (PMC11329638; doi:10.1038/s41598-024-69793-7)
Supplement: Supplementary file 1 — Supplementary Information. [file 41598_2024_69793_MOESM1_ESM.pdf]

Supplementary Information for

Region-selective and site-specific glycation of influenza proteins surrounding the viral envelope membrane

Yi-Min She[1]*, Zongchao Jia[2], Xu Zhang[1,3]*

[1]Centre for Oncology, Radiopharmaceuticals and Research, Biologic and Radiopharmaceutical Drugs Directorate, Health Canada, Ottawa, Ontario K1A 0K9, Canada. [2]Department of Biomedical and Molecular Sciences, Queen's University, Kingston, Ontario K7L 3N6, Canada. [3]School of Pharmaceutical Sciences, Faculty of Medicine, University of Ottawa, Ottawa, Ontario K1H 8M5, Canada.

*Corresponding authors: yi-min.she@hc-sc.gc.ca and xu.zhang@hc-sc.gc.ca

Table of Contents

Figure S1 Mass spectrometric identification of glycation sites in the influenza A/Brisbane/10/2010 …………Page S3
Figure S2 Sequence conservation of matrix 1 of six influenza strains..…………………..…………….……......Page S10
Figure S3 Sequence conservation of hemagglutinins between influenza A/California/7/2009 (H1N1) and A/Brisbane/10/2010 (H1N1-like)………………………………….....…….……….….……...Page S11
Figure S4 Structural locations of antigenic epitopes vs. the glycated lysine sites in the globular head domain of hemagglutinin in influenza A/Brisbane/10/2010…………………………………..……..........…....Page S12
Figure S5 Sequence comparison of hemagglutinin of six influenza vaccine strains……………….……..…...Page S13
Figure S6 Sequence comparison of neuraminidase of six influenza strains………………..……..……..……Page S15
Figure S7 Hemagglutinin peptides containing hexose-modified arginine. …………………..……..……..…...Page S16
Figure S8 False-positive identification of the hexose-glycated arginine of HA and NA peptides.…………...Page S17

Table S1 Identification of glycated peptides in influenza A/Brisbane/10/2010…………………..…….........Page S18
Table S2 List of the glycated peptides and glycation sites of influenza proteins ..……………….................Page S19
Table S3 Motif analysis of sequences surrounding the glycation sites…………..………..………………Page S21
Table S4 Influenza vaccine materials………………………………………….………………..……......Page S23

M1: ^{9}TYVLSIIPSGPL**K**AE^{23} Hexosylation @ Lys21

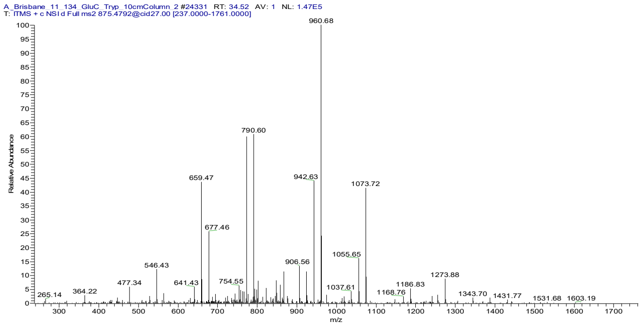

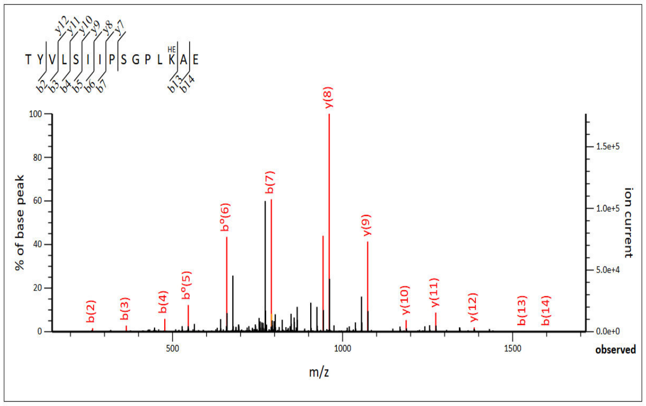

M1: ^{33}AG**K**NTDLEALMEW^{45} Hexosylation @ Lys35

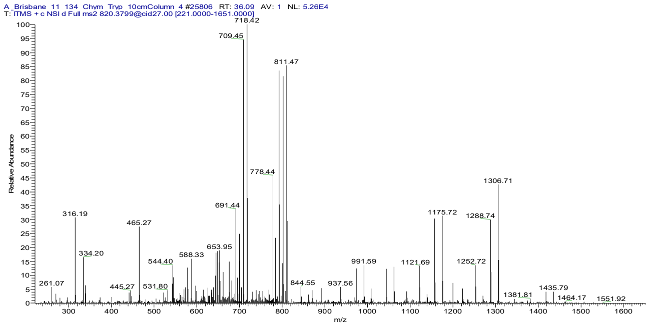

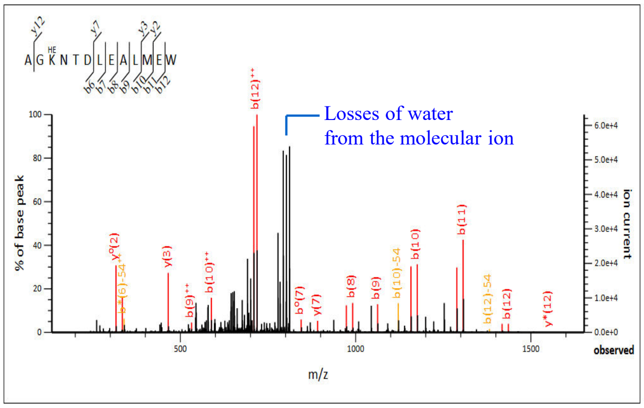

M1: ^{47}**K**TRPILSPL^{55} Hexosylation @ Lys47

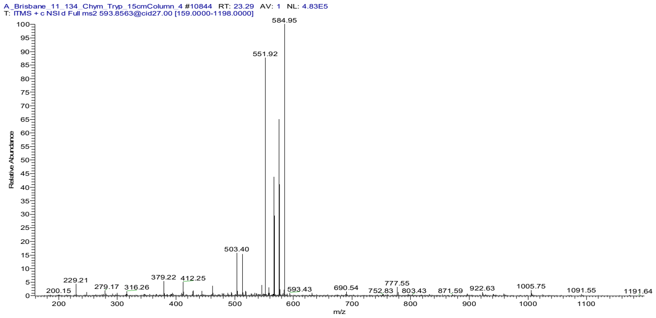

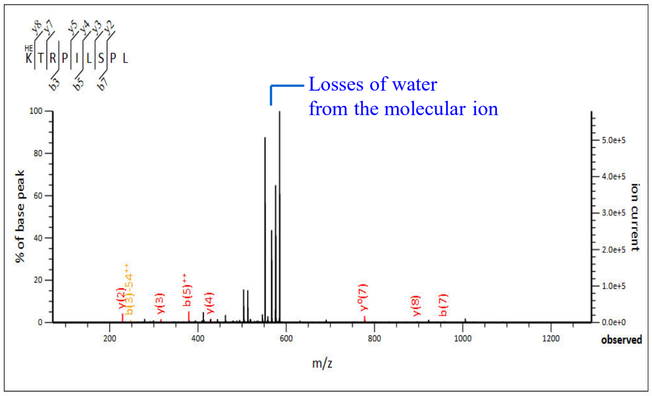

M1: ^{56}T**K**GILGF^{62} Hexosylation @ Lys57

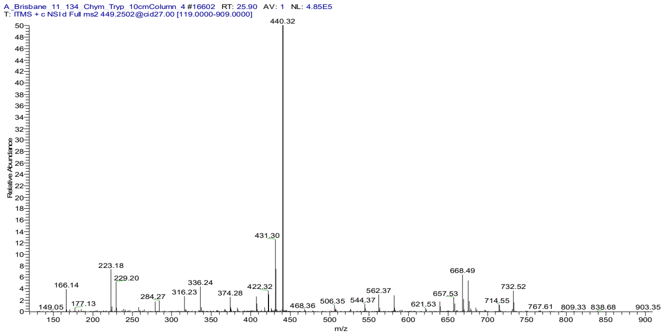

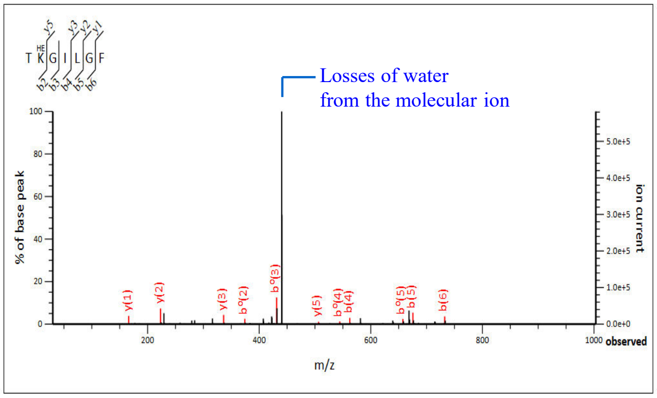

M1: 95-102 ^{95}RAV**K**LYKK^{102} Hexosylation @ Lys98

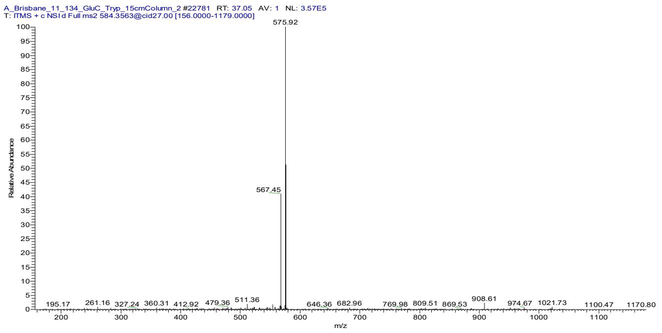

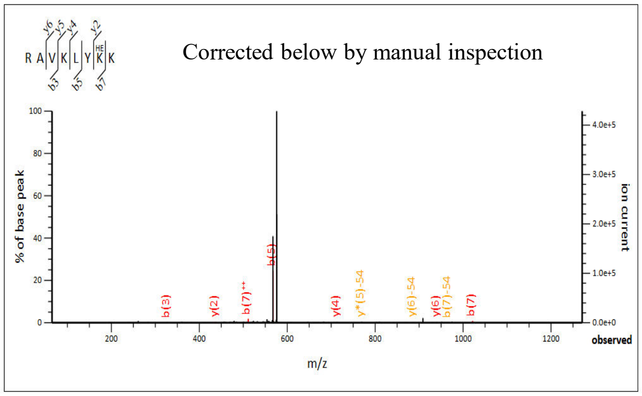

M1: 95-102 ^{95}RAV**K**LYKK^{102} Hexosylation @ Lys98

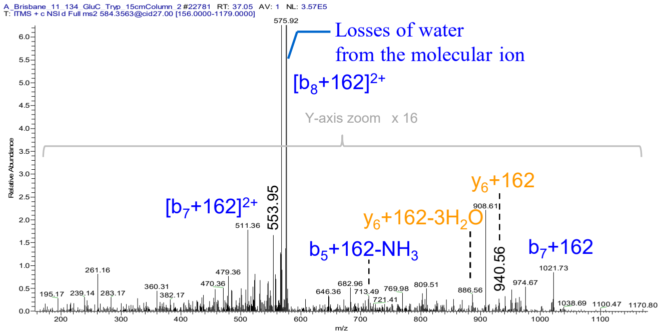

***** **M1:** 96-102 ^{96}AV**K***LYKK^{102}

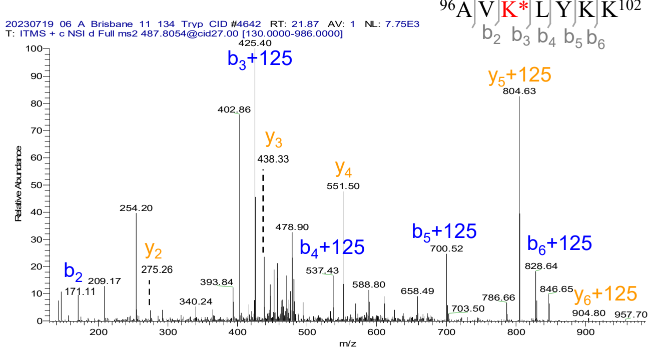

M1: ^{106}EITFHGA**K**EVSLSYSTGALASCMGLIYNR^{134}

Hexosylation @ Lys113

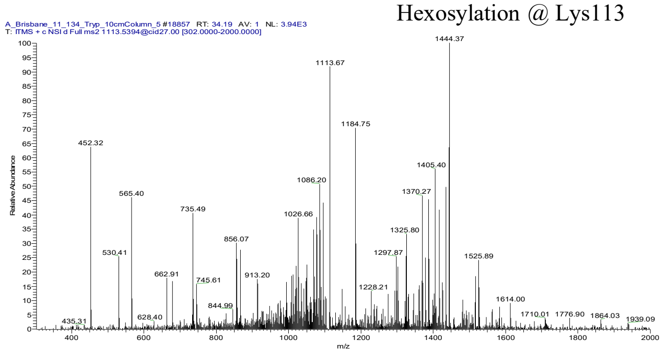

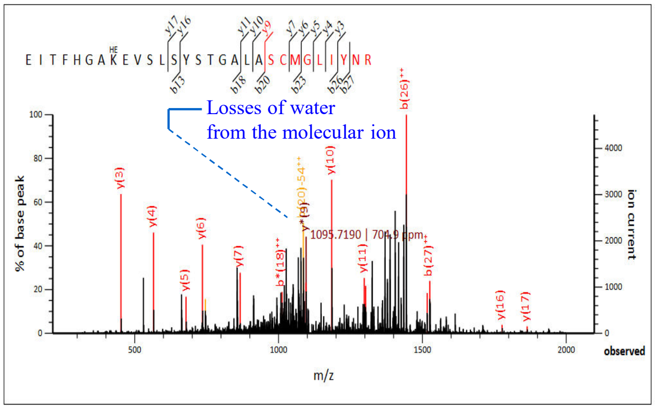

M1: ^{179}MVLASTTA**K**AME^{190} Hexosylation @ Lys187

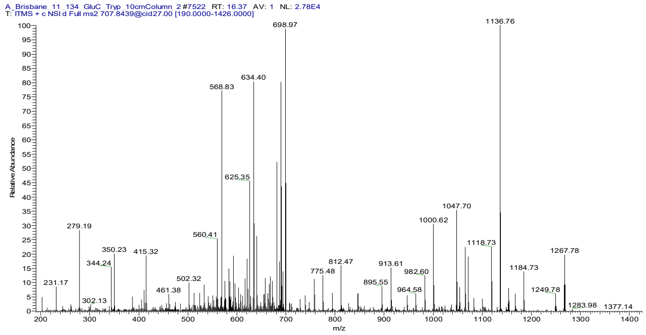

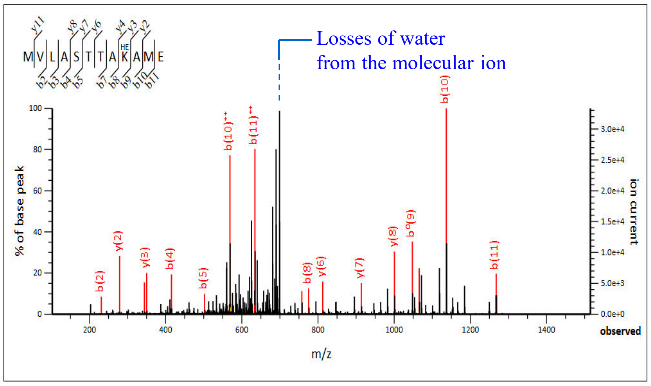

M1: ^{218}TIGTHPSSSAGL**K**DDLLENLQAYQK^{242}

Hexosylation @ Lys230

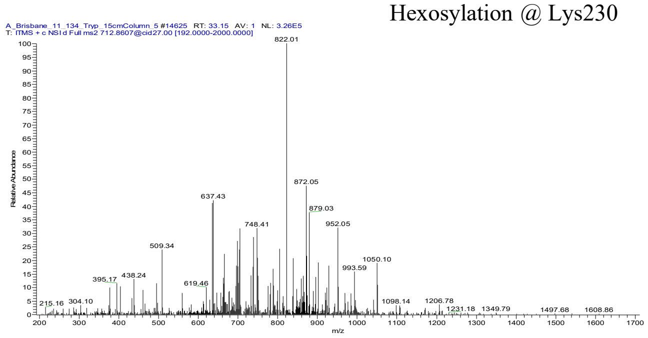

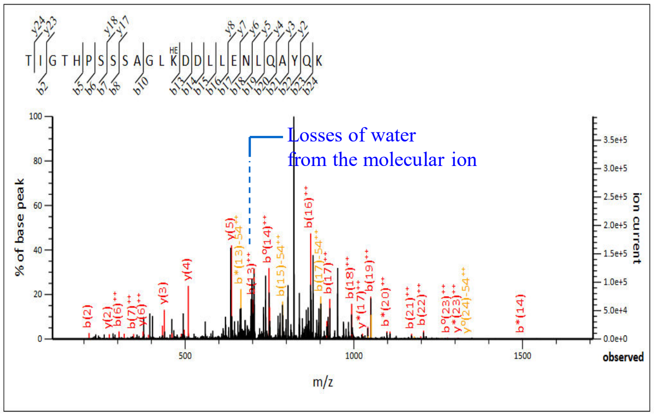

M1: ^{218}TIGTHPSSSAGLKDDLLENLQAYQ**K**R^{243}

Hexosylation @ Lys242

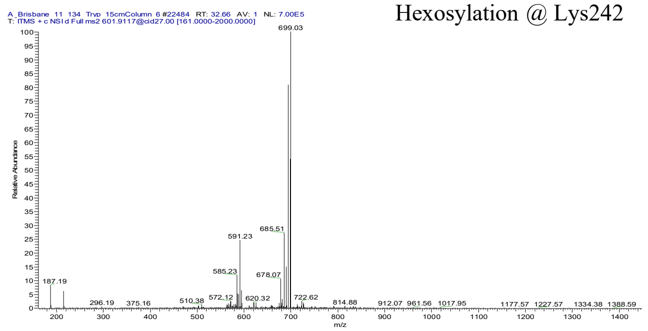

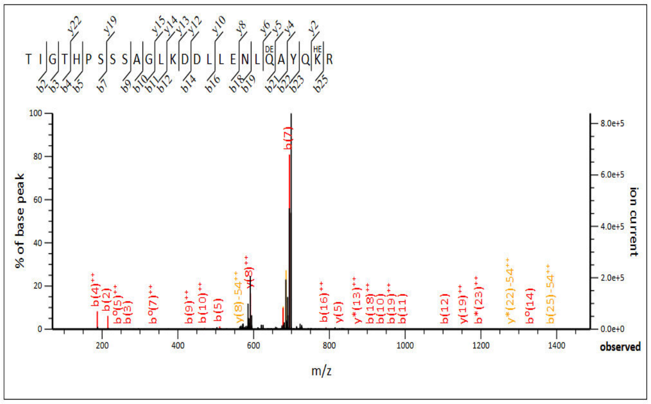

HA: ^{63}GVAPLHLG**K**CNIAGWILGNPE^{83}

Hexosylation @ Lys71

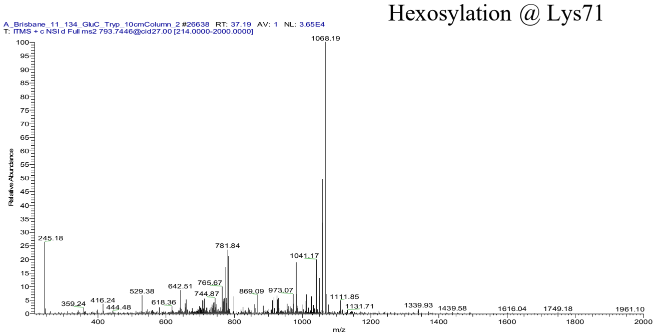

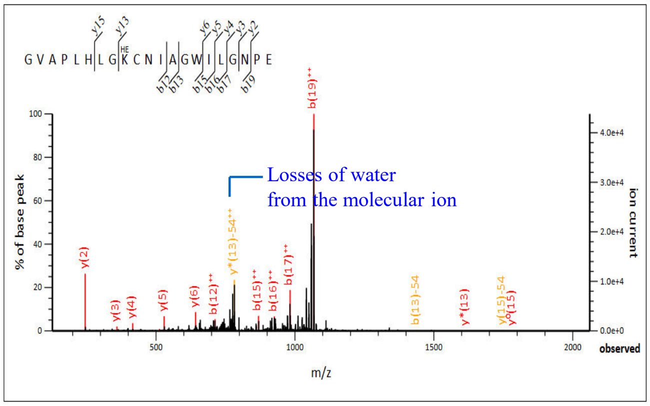

HA: ^{163}**K**NLIW^{167} Hexosylation @ Lys163

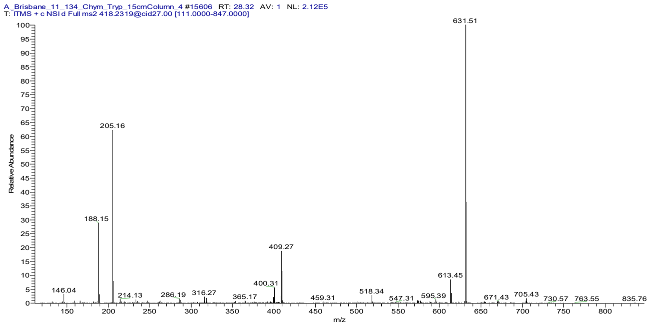

HA: ^{163}**K**NLIW^{167} Hexosylation @ Lys163

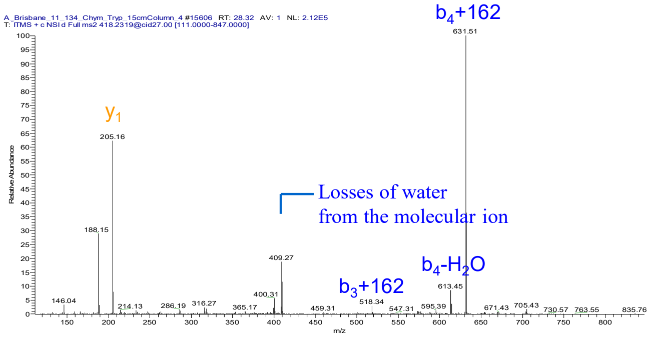

HA: ^{187}G**K**EVLVLW^{194} Hexosylation @ Lys188

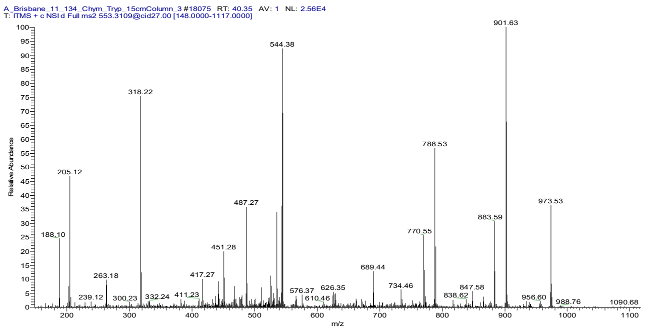

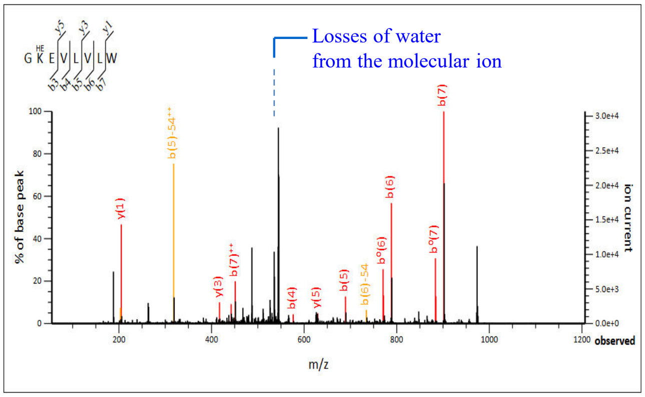

HA: ^{249}TLVEPGD**K**ITF^{259} Hexosylation @ Lys256

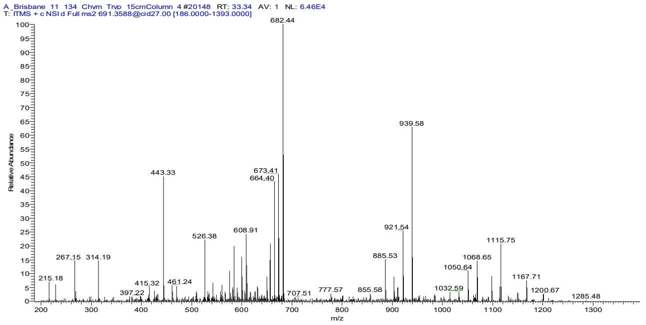

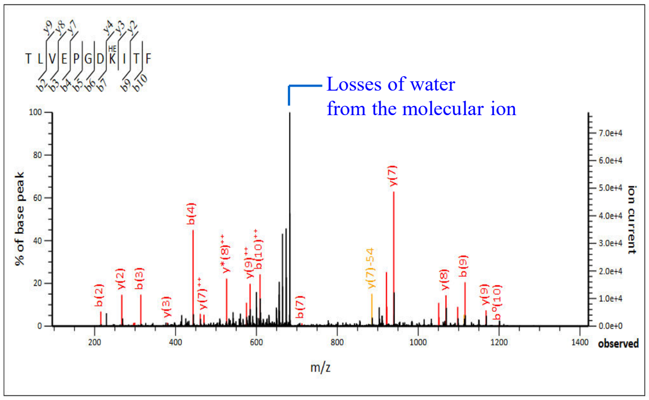

HA: ^{277}NAGSGIIISDTPVHDCNTTCQTP**K**GAINTSLPF^{309}

Hexosylation @ Lys300

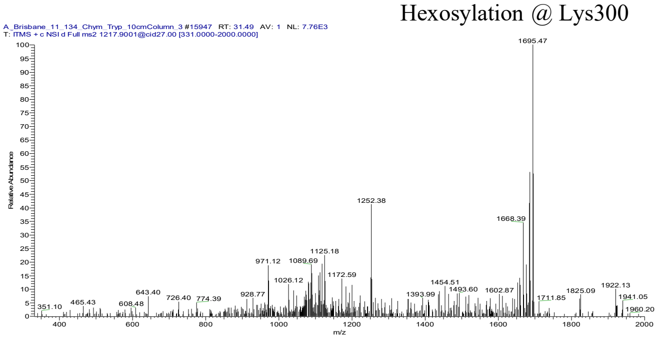

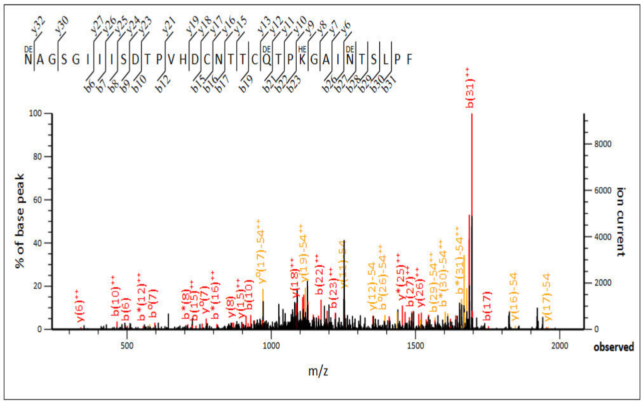

HA: ^{277}NAGSGIIISDTPVHDCNTTCQTP**K**GAINTSLPFQNIHPITIGK^{319}

Hexosylation @ Lys300

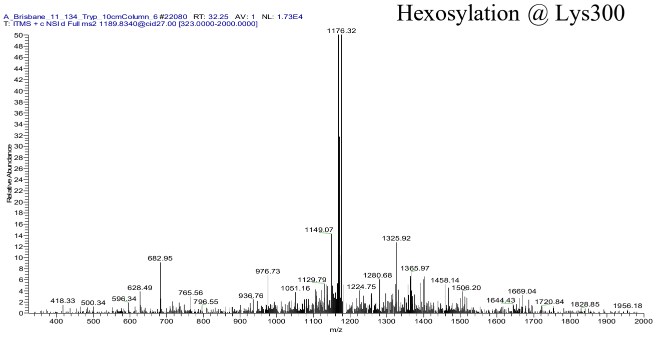

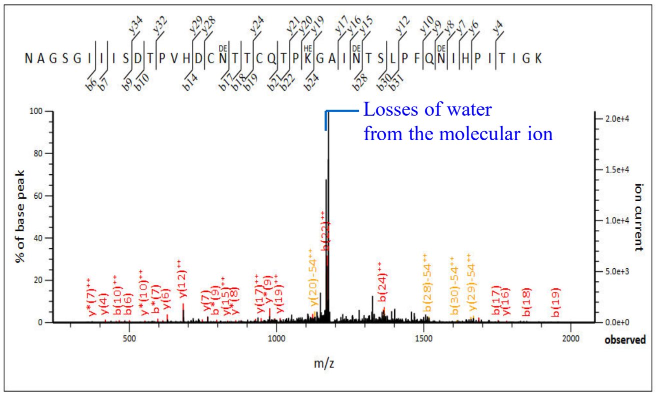

HA: ^{301}GAINTSLPFQNIHPITIG**K**CPK^{322} Hexosylation @ Lys319

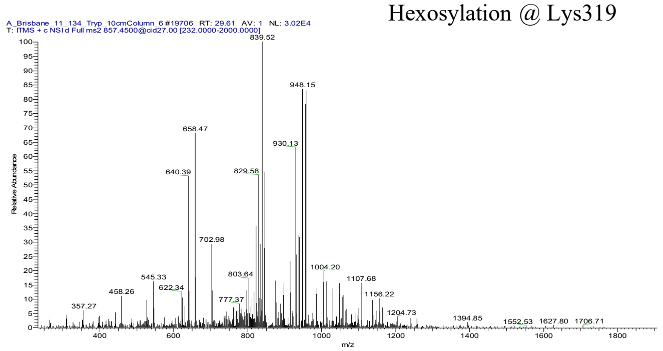

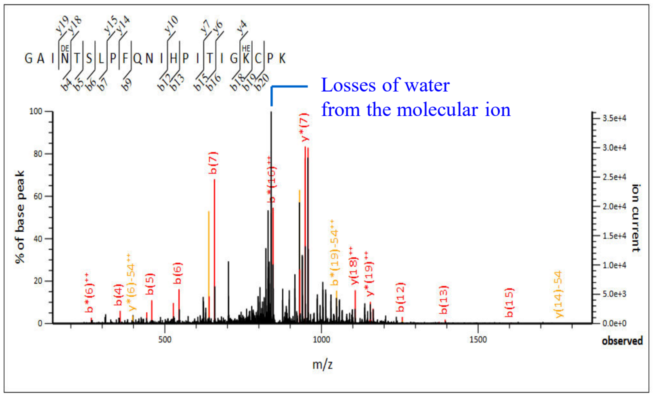

HA: ^{384}STQNAID**K**ITNK^{395} Hexosylation @ Lys391

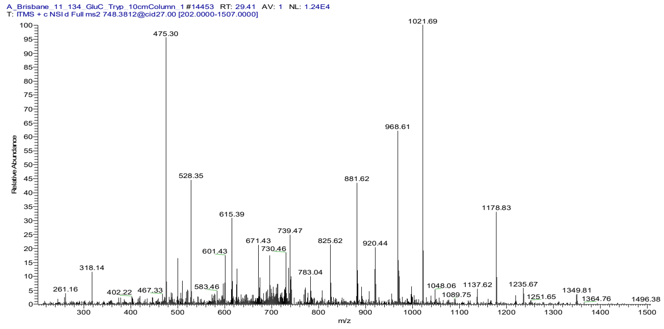

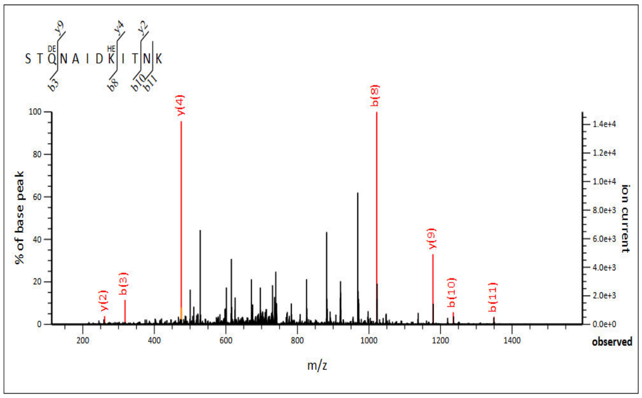

HA: ^{402}**K**MNTQFTAVGK^{412} Hexosylation @ Lys402

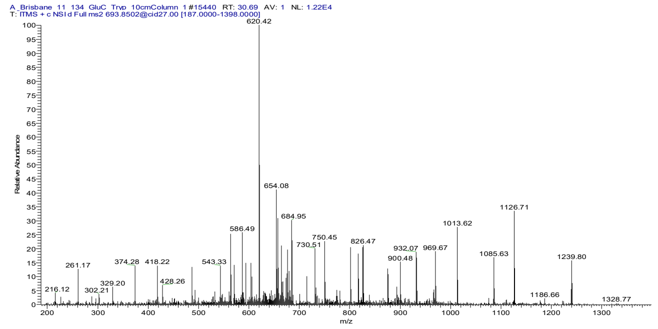

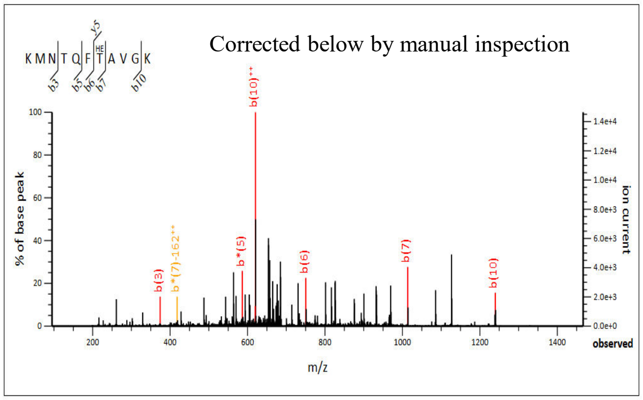

HA: ^{402}**K**MNTQFTAVGK^{412} Hexosylation @ Lys402

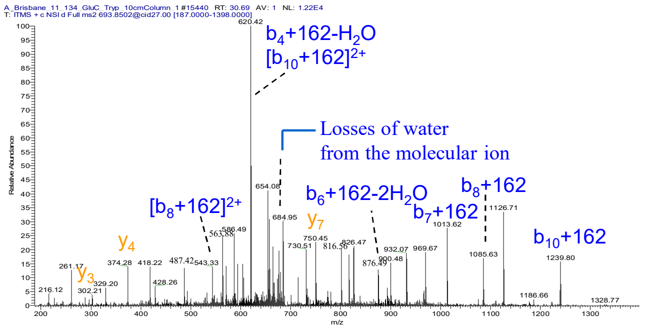

HA: ^{427}**K**VDDGFLDIW^{436} Hexosylation @ Lys427

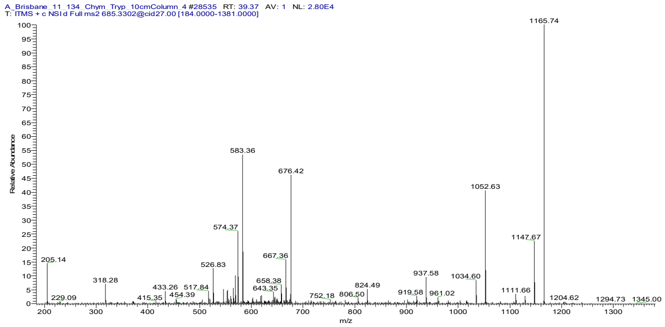

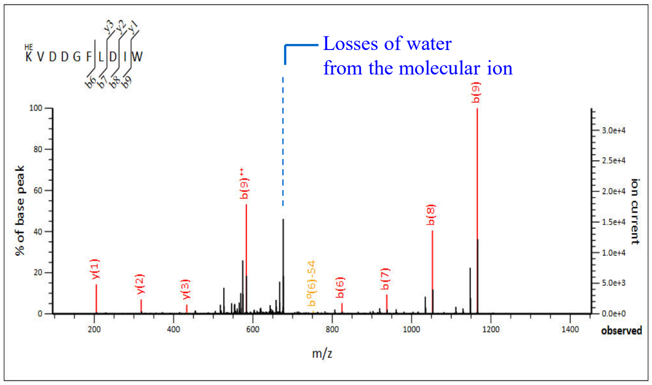

HA: ^{453}DYHDSNV**K**NLY^{463} Hexosylation @ Lys460

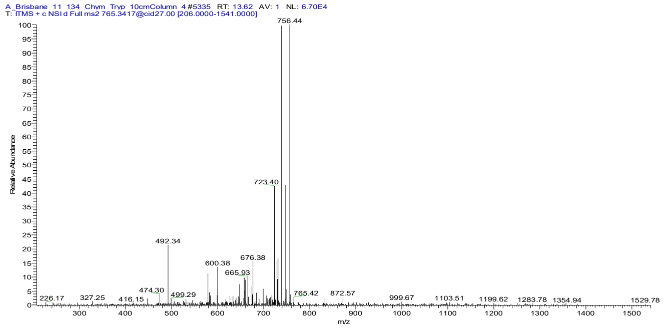

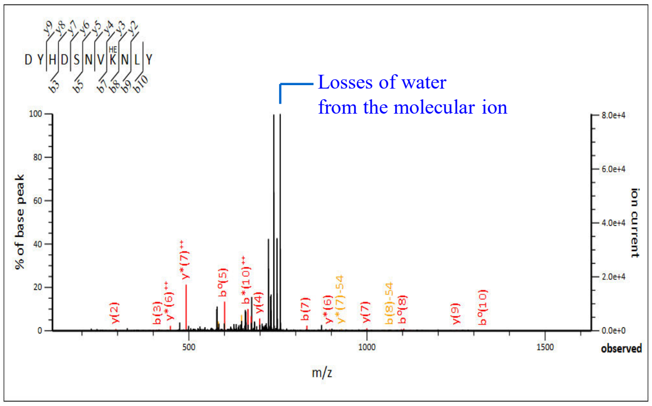

NA: ^{85}LAGNSSLCPVSGWAIYS**K**D^{103} Hexosylation @ Lys102

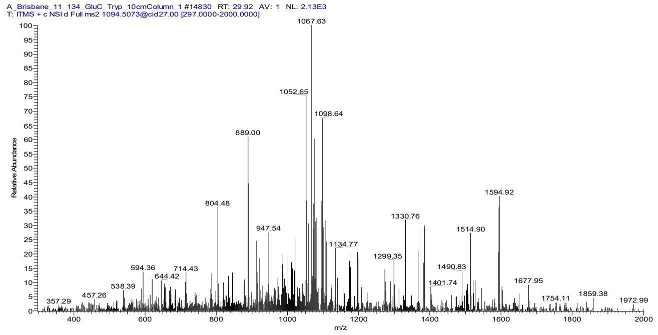

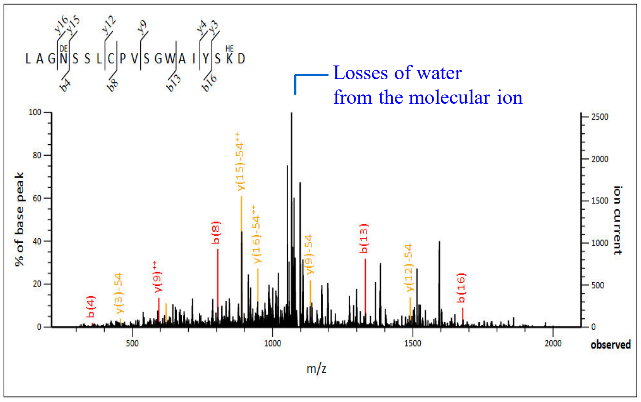

NA: ^{85}LAGNSSLCPVSGWAIYS**K**DNSIR^{107} Hexosylation @ Lys102

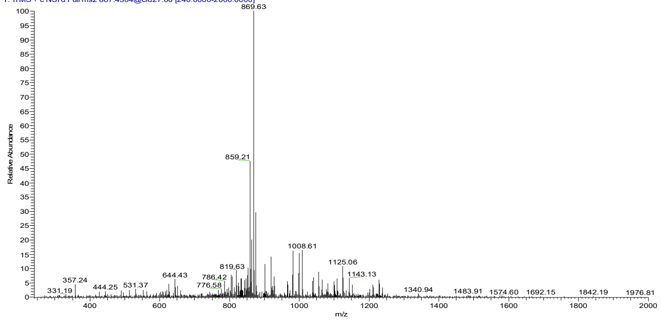

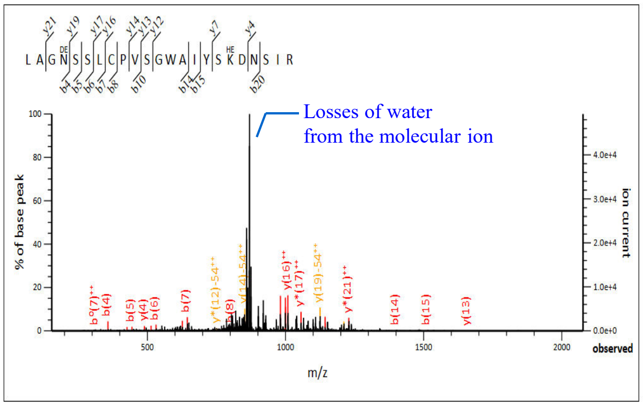

NA: ^{143}**K**HSNGTIK^{150} Hexosylation @ Lys143

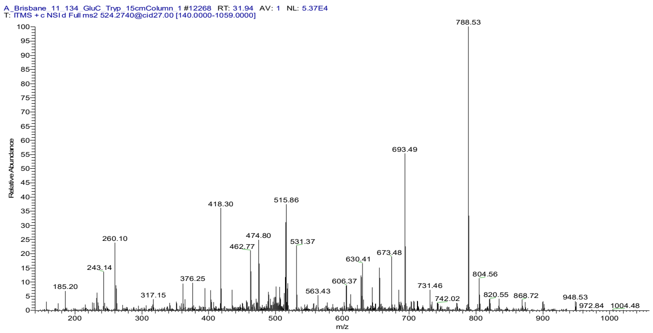

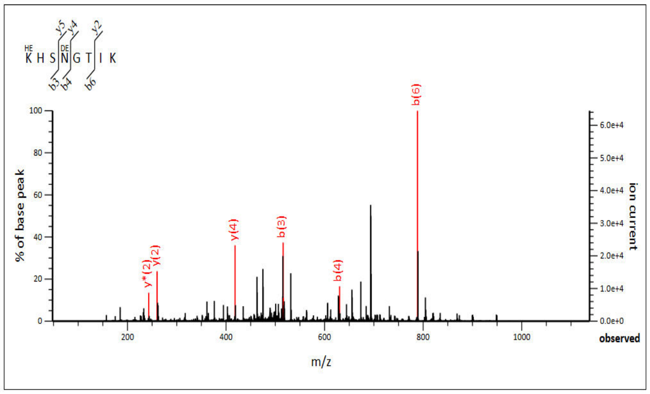

NA: ^{332}TGSCGPVSSNGANGV**K**GF^{349} Hexosylation @ Lys347

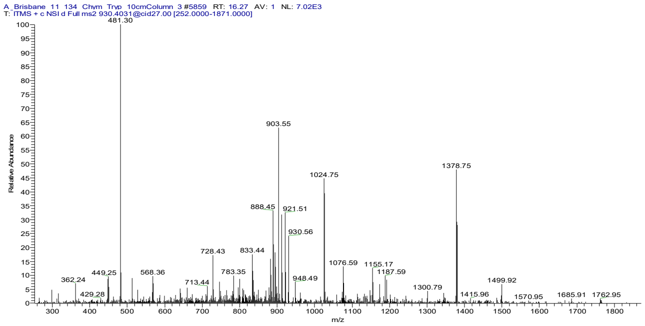

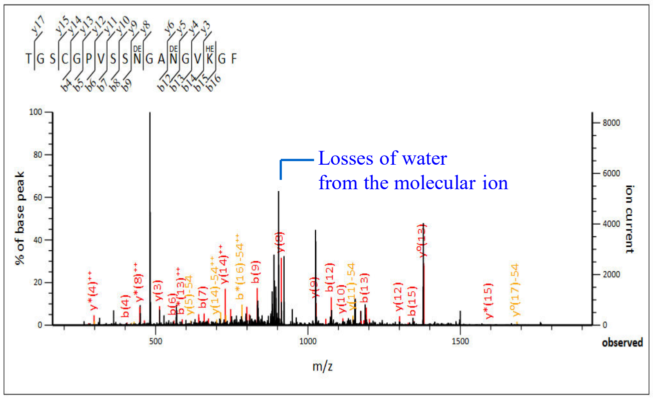

NA: ^{412}LTGLDCI**K**PCFWVE^{425} Hexosylation @ Lys419

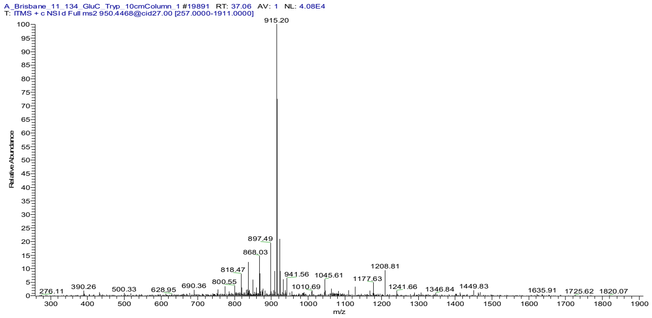

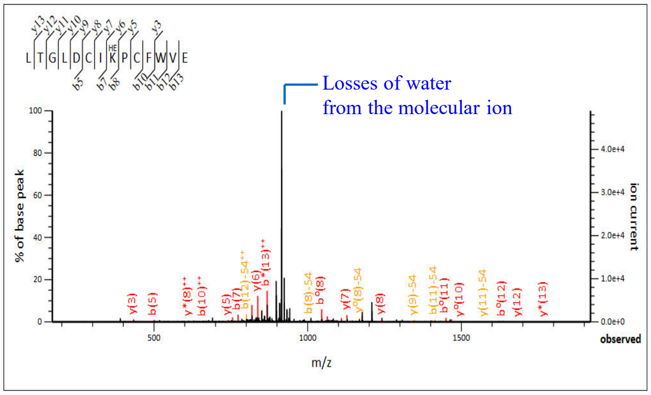

Supplementary figure S1. Mass spectrometric identification of glycation sites in the influenza A/Brisbane/10/2010 by MS/MS sequencing and Mascot database searching. The raw LC MS/MS spectrum is shown on the left panel, and the corresponding search results and manual data inspection are shown on the right panel. HA: hemagglutinin, NA: neuraminidase, M1: matrix protein M1. * In the case of low abundance poor fragmentations of a basic residue–rich peptide, the assignment of the occurring glycation site at Lys98 of M1 has been further verified as the highly reactive residue of the protein. As supporting evidence, Lys98 is found to be the preferred reaction site of peptide 96-102 when N-ethylmaleimide is incorporated in the influenza vaccine, in which the lysine is modified by an increased mass of 125 Da.

```
                                  21              35             47           57
H1N1 MSLLTEVETYVLSIIPSGPLKAEIAQRLESVFAGKNTDLEALMEWLKTRPILSPLTKGIL 60
H3N2 MSLLTEVETYVLSIIPSGPLKAEIAQRLEDVFAGKNTDLEVLMEWLKTRPILSPLTKGIL 60
H5N1 MSLLTEVETYVLSIIPSGPLKAEIAQKLEDVFAGKNTDLEALMEWLKTRPILSPLTKGML 60
H7N9 MSLLTEVETYVLSIIPSGPLKAEIAQRLEDVFAGKNADLEALMEWIKTRPILSPLTKGIL 60
B1   MSLFGDTIAYLLSLTEDGEGKAELAEKLHCWFGGKEFDLDSALEWIKNKRCLTDIQKALI 60
B2   MSLFGDTIAYLLSLTEDGEGKAELAEKLHCWFGGKEFDLDSALEWIKNKRCLTDIQKALI 60
     **:: :. :*:**:  .*  ***:*::*.  *.**: **:  :** *.:  *: : *.::
                                                 98              113
H1N1 GFVFTLTVPSERGLQRRRFVQNALNGNGDPNNMDRAVKL-YKKLKREITFHGAKEVSLSY 119
H3N2 GFVFTLTVPSERGLQRRRFVQNALNGNGDPNNMDKAVKL-YRKLKREITFHGAKEISLSY 119
H5N1 GFVFTLTVPSERGLQRRRFVQNALNGNGDPNNMDRAVKL-YKKLKREITFHGAKEVALSY 119
H7N9 GFVFTLTVPSERGLQRRRFVQNALNGNGDPNNMDKAVKL-YKKLKREMTFHGAKEVALSY 119
B1   GASICFLKPKDQE-RKRRFITEPLSGMGTTATKKKGLILAERKMRRCVSFHEAFEIAEGH 119
B2   GASICFLKPKDQE-RKRRFITEPLSGMGTTATKKKGLILAERKMRKCVSFHEAFEIAEGH 119
     *  : :  *.::  ::***: : *.* *    . .:.: *  :*::: ::** * *:: .:

H1N1 STGALASCMGLIYNRMGTVTTEAAFGLVCATCEQIADSQHRSHRQMATTTNPLIRHENRM 179
H3N2 SAGALASCMGLIYNRMGAVTTEVAFGLVCATCEQIADSQHRSHRQMVTTTNPLIRHENRM 179
H5N1 STGALASCMGLIYNRMGTVTTEVAFGLVCATCEQIADSQHRSHRQMATITNPLIRHENRM 179
H7N9 STGALASCMGLIYNRMGTVTAEGALGLVCATCEQIADAQHRSHRQMATTTNPLIRHENRM 179
B1   ESSALLYCLMVMYLNPGNYSMQVKLGTLCALCEKQASHSHRAHSRAARSSVPGVRREMQM 179
B2   ESSALLYCLMVMYLNPGNYSMQVKLGTLCALCEKQASHSHRAHSRAARSSVPGVRREMQM 179
     .:.**  *: ::* . *  : :  :* :** **: *. .**:* : .  : * :*:* :*
          187                                           230
H1N1 VLASTTAKAMEQMAGSSEQAAEAMEVANQTRQMVHAMRTIGTHPSSSAGLKDDLLENLQA 239
H3N2 VLASTTAKAMEQMAGSSEQAAEAMEVASQARQMVQAMRTIGTHPSSSAGLKNDLLENLQA 239
H5N1 VLASTTAKAMEQMAGSSEQAAEAMEVANQARQMVQAMRTIGTHPNSSAGLRDNLLENLQA 239
H7N9 VLASTTAKAMEQMAGSSEQAAEAMEVASQARQMVQAMRTVGTHPNSSTGLKDDLIENLQA 239
B1   VSAMNTAKTMNGMGKGEDVQK----LAEELQSNIGVLRSLGASQKNGEGIAKDVMEVLKQ 235
B2   VSAMNTAKTMNGMGKGEDVQK----LAEELQSNIGVLRSLGASQKNGEGIAKDVMEVLKQ 235
     * * .***:*: *. ..:       :*.: :. : .:*::*:   ... *: .:::* *:
   242
H1N1 YQKRMGVQMQRFK 252
H3N2 YQKRMGVQMQRFK 252
H5N1 YQKRMGVQMQRFK 252
H7N9 YQNRMGVQLQRFK 252
B1   SSMGNSALVKKYL 248
B2   SSMGNSALVKKYL 248
      .   .. ::::
```

Supplementary figure S2. Sequence conservation of matrix 1 of six influenza strains. Multiple sequence alignment was performed using Clustal Omega (https://www.ebi.ac.uk/Tools/msa/clustalo/).
H1N1: A/Brisbane/10/2010 (GSAID accession no. EPI745534)
H3N2: A/Hong Kong/4801/2014 (GSAID accession no. EPI614409)
H5N1: A/turkey/Turkey/1/2005 (GSAID accession no. EPI118789)
H7N9: A/Anhui/1/2013 (GSAID accession no. EPI439506)
Influenza B1: B/Brisbane/60/2008 (GSAID accession no. EPI366462)
Influenza B2: B/Phuket/3073/2013 (GSAID accession no. EPI1649067)

```
CA2009   MKAILVVLLYTFATANADTLCIGYHANNSTDTVDTVLEKNVTVTHSVNLLEDKHNGKLCK 60
BRI2010  MKAILVVLLYTFATANADTLCIGYHANNSTDTVDTVLEKNVTVTHSVNLLEDKHNGKLCK 60
         ************************************************************
                                                                       Cb sites
CA2009   LRGVAPLHLGKCNIAGWILGNPECESLSTASSWSYIVETPSSDNGTCYPGDFIDYEELRE 120
BRI2010  LRGVAPLHLGKCNIAGWILGNPECESLSTASSWSYIVETSSSDNGTCYPGDFIDYEELRE 120
         *************************************** ********************
                                                                       Sa sites
CA2009   QLSSVSSFERFEIFPKTSSWPNHDSNKGVTAACPHAGAKSFYKNLIWLVKKGNSYPKLSK 180
BRI2010  QLSSVSSFERFEIFPKTSSWPDHDSNKGVTAACPHAGAKSFYKNLIWLVKKGNSYPKLSK 180
         *********************:**************************************
                                                                       Ca sites
CA2009   SYINDKGKEVLVLWGIHHPSTSADQQSLYQNADAYVFVGSSRYSKKFKPEIAIRPKVRDQ 240
BRI2010  SYINDKGKEVLVLWGIHHPSTSADQQSLYQNADAYVFVGTSRYSKKFKPEIAIRPKVRDQ 240
         ***************************************:********************
                                                                       Sb sites
CA2009   EGRMNYYWTLVEPGDKITFEATGNLVVPRYAFAMERNAGSGIIISDTPVHDCNTTCQTPK 300
BRI2010  EGRMNYYWTLVEPGDKITFEATGNLVVPRYAFAMERNAGSGIIISDTPVHDCNTTCQTPK 300
         ************************************************************

CA2009   GAINTSLPFQNIHPITIGKCPKYVKSTKLRLATGLRNIPSIQSRGLFGAIAGFIEGGWTG 360
BRI2010  GAINTSLPFQNIHPITIGKCPKYVKSTKLRLATGLRNVPSIQSRGLFGAIAGFIEGGWTG 360
         *************************************:**********************

CA2009   MVDGWYGYHHQNEQGSGYAADLKSTQNAIDEITNKVNSVIEKMNTQFTAVGKEFNHLEKR 420
BRI2010  MVDGWYGYHHQNEQGSGYAADLKSTQNAIDKITNKVNSVIEKMNTQFTAVGKEFNHLEKR 420
         ******************************:*****************************
CA2009   IENLNKKVDDGFLDIWTYNAELLVLLENERTLDYHDSNVKNLYEKVRSQLKNNAKEIGNG 480
BRI2010  IENLNKKVDDGFLDIWTYNAELLVLLENERTLDYHDSNVKNLYEKVRSQLKNNAKEIGNG 480
         ************************************************************

CA2009   CFEFYHKCDNTCMESVKNGTYDYPKYSEEAKLNREEIDGVKLESTRIYQILAIYSTVASS 540
BRI2010  CFEFYHKCDNTCMESVKNGTYDYPKYSEEAKLNREEIDGVKLESTRIYQILAIYSTVASS 540
         ************************************************************

CA2009   LVLVVSLGAISFWMCSNGSLQCRICI           566
BRI2010  LVLVVSLGAISFWMCSNGSLQCRICI           566
         **************************
```

Supplementary figure S3. Sequence conservation of hemagglutinins between influenza A/California/7/2009 (H1N1) and A/Brisbane/10/2010 (H1N1-like). Multiple sequence alignment shows the amino acid identity of over 99%. Abbreviations: CA2009, A/California/7/2009 strain (GSAID accession no. EPI1593569); BRI2010, A/Brisbane/10/2010 strain (GSAID accession no. EPI745532). The antigenic sites are coloured according to the previous report by Xu R. et al.: Sa site in magenta, Sb site in cyan; Ca site in orange; and Cb site in blue.

Reference: Xu R, Ekiert DC, Krause JC, Hai R, Crowe JE Jr, Wilson IA. Structural basis of preexisting immunity to the 2009 H1N1 pandemic influenza virus. *Science* **328**, 357-360 (2010).

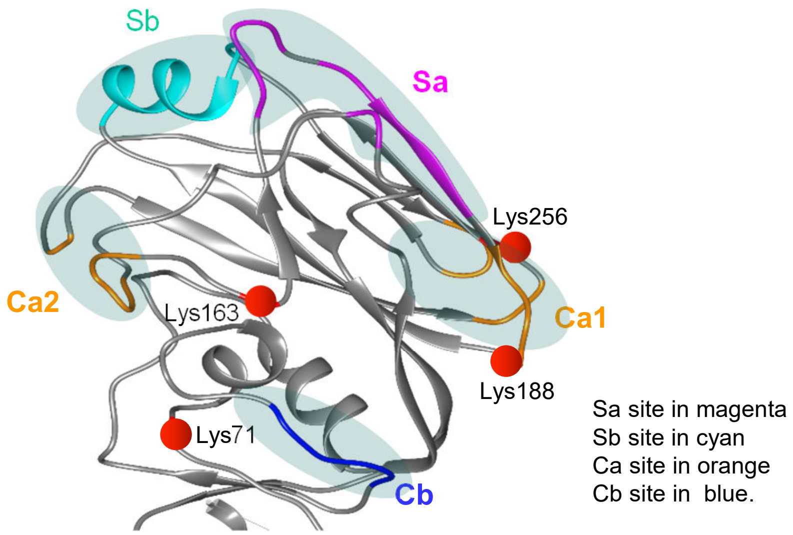

Supplementary figure S4. Structural locations of antigenic epitopes *vs.* the glycated lysine sites at the globular head domain of hemagglutinin in influenza A/Brisbane/10/2010. The antigenic sites of Sa, Sb, Ca and Cb are coloured as indicated in the sequence regions (see Fig. S3) surrounding the receptor-binding pocket. The glycated residues of Lys71, Lys163, Lys188 and Lys256 are highlighted in red.

```
H1N1 MKA------ILVVLLYTF---ATANADTLCIGYHANNSTDTVDTVLEKNVTVTHSVNLLE 51
H3N2 MKTIIALSYILCLVFAQKIPGNDNSTATLCLGHHAVPNGTIVKTITNDRIEVTNATELVQ 60
H5N1 ME-------KIVLLLAIV---SLVKSDQICIGYHANNSTEQVDTIMEKNVTVTHAQDILE 50
H7N9 MNT-----QILVFALIAIIP---TNADKICLGHHAVSNGTKVNTLTERGVEVVNATETVE 52
B1   MKA------IIVLLMV-----VTSNADRICTGITSSNSPHVVKTATQGEVNVTGVIPLTT 49
B2   MKA------IIVLLMV-----VTSNADRICTGITSSNSPHVVKTATQGEVNVTGVIPLTT 49
     *:        : . :         .:  :* *  :  .   *.*  :  : *.

H1N1 DKHNGKLCKL-----RGVAPLHLGKCNIAGWILGNPECESLSTASSWSYIVETSSSDNGT 106
H3N2 NSSIGEICDS------PHQILDGENCTLIDALLGDPQCDGFQNK-KWDLFVERS-KAYSS 112
H5N1 KTHNGKLCDL-----DGVKPLILRDCSVAGWLLGNPMCDEFLNVPEWSYIVEKINPANDL 105
H7N9 RTNIPRICSK------GKRTVDLGQCGLLGTITGPPQCDQFLEF-SADLIIERR-EGSDV 104
B1   TPTKSHFANLKGTETRGKLCPKCLNCTDLDVALGRPKCTGKIPSARVSILHEVR-PVTSG 108
B2   TPTKSYFANLKGTRTRGKLCPDCLNCTDLDVALGRPMCVGTTPSAKASILHEVR-PVTSG 108
          :..                .*   .   * * *         . : *      .

H1N1 CYPGDFIDYEELREQLSSVSSFERFEIFP----KTSSWPDHDSNKGVTAACPH-AGAKSF 161
H3N2 CYPYDVPDYASLRSLVASSGTLEFNN-------ESFNWTGV-TQNGTSSACIR-RSSSSF 163
H5N1 CYPGNFNDYEELKHLLSRINHFEKIQIIP----K-SSWSDHEASAGVSSACPY-QGRSSF 159
H7N9 CYPGKFVNEEALRQILRESGGIDKEA-------MGFTYSGI-RTNGATSACR--RSGSSF 154
B1   CFPIMH-DRTKIRQLPNLLRGYEHIRLSTHNVINAENAPGGPYKIGTSGSCPNITNGNGF 167
B2   CFPIMH-DRTKIRQLPNLLRGYEKIRLSTQNVIDAEKAPGGPYRLGTSGSCPNATSKIGF 167
     *:*    :   ::         :             .  .     *.:.:*    .  .*

H1N1 YKNLIWLVKK--GNSY----PKLSKSYINDKGKEVLVLWGIHHPSTSADQQSLYQNADAY 215
H3N2 FSRLNWLTHL--NYKY----PALNVTMPNNEQFDKLYIWGVHHPGTDKDQIFPYAQSSGR 217
H5N1 FRNVVWLIKK--DNAY----PTIKRSYNNTNQEDLLVLWGIHHPNDAAEQTRLYQNPTTY 213
H7N9 YAEMKWLLSNTDNAAF----PQMTKSYKNTRKSPALIVWGIHHSVSTAEQTKLYGSGNKL 210
B1   FATMAWAVPKNDKNKTATNPLTIEVPYICTEGEDQITVWGFHSDDE-TQMAKLYGDSKPQ 226
B2   FATMAWAVPKDN-YKNATNPLTVEVPYICTEGEDQITVWGFHSDNK-TQMKSLYGDSNPQ 225
     :  : *                :       .    : :**.*      :    * .

H1N1 VFVGT-SRYSKKFKPEIAIRPKV-----RDQEGRMNYYWTLVEPGDKITFEATGNLVVPR 269
H3N2 IFVST-KRSQQAVIPNIGSRPRI-----RDIPSRISIYWTIVKPGDILLINSTGNLIAPR 271
H5N1 ISVGT-STLNQRLVPKIATRSKV-----NGQSGRMEFFWTILKPNDAINFESNGNFIAPE 267
H7N9 VTVGS-SNYQQSFVPSPGARPQV-----NGLSGRIDFHWLMLNPNDTVTFSFNGAFIAPD 264
B1   KFTSSANGVTTHYVSQIGGFPNQTEDGGLPQSGRIVVDYMVQKSGKTGTITYQRGILLPQ 286
B2   KFTSSANGVTTHYVSQIGDFPDQTEDGGLPQSGRIVVDYMMQKPGKTGTIVYQRGVLLPQ 285
       ..: .        . .             .*:    : : : ..   :     .: *

H1N1 YAFAMERNAGSGIIISDTPVHDCNTTCQ-TPKGAINTSLPF-QNIHPITIGKCPKYVKST 327
H3N2 GYFKI-RSGKSSIMRSDAPIGKCKSECI-TPNGSIPNDKPF-QNVNRITYGACPRYVKHS 328
H5N1 NAYKIVKKGDSTIMKSELEYGNCNTKCQ-TPIGAINSSMPF-HNIHPLTIGECPKYVKSS 325
H7N9 RASFL-RGKSMGIQSGVQVDANCEGDCY-HSGGTIISNLPF-QNIDSRAVGKCPRYVKQR 321
B1   KVWCA--SGRSKVIKGSLPLIGE-ADCLHEKYGGLNKSKPYYTGEHAKAIGNCPIWVKT- 342
B2   KVWCA--SGRSKVIKGSLPLIGE-ADCLHEEYGGLNKSKPYYTGKHAKAIGNCPIWVKT- 341
                  : .          *     * : .. *:  . .  : * ** :**
```

Supplementary figure S5. Sequence comparison of hemagglutinin of six influenza vaccine strains.
H1N1: A/Brisbane/10/2010 (GSAID accession no. EPI745532); H3N2: A/Hong Kong/4801/2014 (GSAID accession no. EPI614414); H5N1: A/turkey/Turkey/1/2005 (GSAID accession no. EPI118794); H7N9: A/Anhui/1/2013 (GSAID accession no. EPI439507); Influenza B1: B/Brisbane/60/2008 (GSAID accession no. EPI2082061); Influenza B2: B/Phuket/3073/2013 (GSAID accession no. EPI1649072)

```
                      Conserved fusion peptide
H1N1 KLRLATGLRNVPSI----QSRGLFGAIAGFIEGGWTGMVDGWYGYHHQNEQGSGYAADLK 383
H3N2 TLKLATGMRNVPEK----QTRGIFGAIAGFIENGWEGMVDGWYGFRHQNSEGRGQAADLK 384
H5N1 RLVLATGLRNSPQGERRRKKRGLFGAIAGFIEGGWQGMVDGWYGYHHSNEQGSGYAADKE 385
H7N9 SLLLATGMKNVPEIP---KGRGLFGAIAGFIENGWEGLIDGWYGFRHQNAQGEGTAADYK 378
B1   PLKLANGTKYRPPA-KLLKERGFFGAIAGFLEGGWEGMIAGWHGYTSHGAHGVAVAADLK 401
B2   PLKLANGTKYRPPA-KLLKERGFFGAIAGFLEGGWEGMIAGWHGYTSHGAHGVAVAADLK 400
      * **.* :  *      : **:*******:*.** *:: **:*:   . .* . *** :

H1N1 STQNAIDKITNKVNSVIEKMNTQFTAVGKEFNHLEKRIENLNKKVDDGFLDIWTYNAELL 443
H3N2 STQAAIDQINGKLNRLIGKTNEKFHQIEKEFSEVEGRIQDLEKYVEDTKIDLWSYNAELL 444
H5N1 STQKAIDGVTNKVNSIIDKMNTQFEAVGREFNNLERRIENLNKKMEDGFLDVWTYNAELL 445
H7N9 STQSAIDQITGKLNRLIEKTNQQFELIDNEFNEVEKQIGNVINWTRDSITEVWSYNAELL 438
B1   STQEAINKITKNLNSLSELEVKNLQRLSGAMDELHNEILELDEKVDDLRADTISSQIELA 461
B2   STQEAINKITKNLNSLSELEVKNLQRLSGAMDELHNEILELDEKVDDLRADTISSQIELA 460
     *** **: :. ::* :      ::   :   :..:. .* :: :    *    :  : : **

H1N1 VLLENERTLDYHDSNVKNLYEKVRSQLKNNAKEIGNGCFEFYHKCDNTCMESVKNGTYDY 503
H3N2 VALENQHTIDLTDSEMNKLFEKTKKQLRENAEDMGNGCFKIYHKCDNACIGSIRNGTYDH 504
H5N1 VLMENERTLDFHDSNVKNLYDKVRLQLRDNAKELGNGCFEFYHRCDNECMESVRNGTYDY 505
H7N9 VAMENQHTIDLADSEMDKLYERVKRQLRENAEEDGTGCFEIFHKCDDDCMASIRNNTYDH 498
B1   VLLSNEGIINSEDEHLLALERKLKKMLGPSAVEIGNGCFETKHKCNQTCLDRIAAGTFDA 521
B2   VLLSNEGIINSEDEHLLALERKLKKMLGPSAVDIGNGCFETKHKCNQTCLDRIAAGTFNA 520
     * :.*:  ::  *..:  *   : :   *   .* : *.***:  *:*:: *:   :   .*::

H1N1 PKYSEEAKLNREEIDGVKLE--STRIYQILAIYSTVASSLVLVVSLGAISFWMCSNGSLQ 561
H3N2 NVYRDEALNNRFQIKGVELK--SGYKDWI-LWISFAISCFLLCVALLGFIMWACQKGNIR 561
H5N1 PQYSEEARLKREEISGVKLE--SIGTYQILSIYSTVASSLALAIMVAGLSLWMCSNGSLQ 563
H7N9 SKYREEAMQNRIQIDPVKLS--SGYKDVI-LWFSFGASCFILLAIVMGLVFICVKNGNMR 555
B1   GEFSLPTFD-SLNITAASLNDDGLDNHTILLYYSTAASSLAVTLMIAIFVVYMVSRDNVS 580
B2   GEFSLPTFD-SLNITAASLNDDGLDNHTILLYYSTAASSLAVTLMLAIFIVYMVSRDNVS 579
       :   :     :*  ..*.  .     *    *   *.: :   :  : .   ....:

H1N1 CRICI 566
H3N2 CNICI 566
H5N1 CRICI 568
H7N9 CTICI 560
B1   CSICL 585
B2   CSICL 584
     * **:
```

Supplementary figure S5 (continued). Sequence comparison of hemagglutinin of six influenza vaccine strains**.** H1N1: A/Brisbane/10/2010 (GSAID accession no. EPI745532); H3N2: A/Hong Kong/4801/2014 (GSAID accession no. EPI614414); H5N1: A/turkey/Turkey/1/2005 (GSAID accession no. EPI118794); H7N9: A/Anhui/1/2013 (GSAID accession no. EPI439507); Influenza B1: B/Brisbane/60/2008 (GSAID accession no. EPI2082061); Influenza B2: B/Phuket/3073/2013 (GSAID accession no. EPI1649072)

```
H1N1 MNPNQKIITIGSVCITIGMANLILQIGNI-IS-------------IWISHSIQLG----N 42
H3N2 MNPNQKIITIGSVSLTISTICFFMQIAIL-IT-------------TVTLHFKQYEFNSPP 46
H5N1 MNPNQKIITIGSICMVIGIVSLMLQIGNM-IS-------------IWVSHSIQTG----N 42
H7N9 MNPNQKILCTSATAIIIGAIAVLIGIANL-GL-------------NIGLHLKPGCNCSHS 46
B1   MLP-----------STIQTLTLFLTSGGVLLSLYVSASLSYLLYSDILLKFSPTEITAPT 49
B2   MLP-----------STIQTLTLFLTSGGVLLSLYVSASLSYLLYSDILLKFSRTEVTAPI 49
     * *             *    .::  . :                    :
H1N1 QNQIETCNQSVITYENNTWVNQTYVNISNTNFAAGQSVV------SV-KLAGNSSLCPVS 95
H3N2 NNQVMLCEPTIIERNITEIV-----YLTNTTIEKEICPK------PAEYRNWSKPQCGIT 95
H5N1 QCQAEP--------------------ISNTKFLTEKAVA------SV-TLAGNSSLCPIS 75
H7N9 QPETTNTSQTIINNYYN-ET-----NITNIQME---ERT------SRNFNNLTKGLCTIN 91
B1   MPL--DCA-----------------NASNVQAVNRSATKGVTLLLPEPEWTYPRLSCPGS 90
B2   MPL--DCA-----------------NASNVQAVNRSATKGVTPLLPEPEWTYPRLSCPGS 90
                                :*                           *  .
          102                                         143
H1N1 GWAIYSKDNSIRI---GSKGDVFVIREPFISCSPLECRTFFLTQGALLNDKHSNGTIKDR 152
H3N2 GFAPFSKDNSIRL---SAGGDIWVTREPYVSCDPDKCYQFALGQGTTLNNVHSNNTVRDR 152
H5N1 GWAVYSKDNSIRI---GSRGDVFVIREPFISCSHLECRTFFLTQGALLNDKHSNGTVKDR 132
H7N9 SWHIYGKDNAVRI---GESSDVLVTREPYVSCDPDECRFYALSQGTTIRGKHSNGTIHDR 148
B1   TFQKALLISPHRFGETKGNSAPLIIREPFIACGPNECKHFALTHYAAQPGGYYNGTRGDR 150
B2   TFQKALLISPHRFGETKGNSAPLIIREPFIACGPKECKHFALTHYAAQPGGYYNGTREDR 150
      :      .  *:      .   : ***:::*.  :*  : * : :   . : *.*  **
H1N1 SPYRTLMSCPIGEVPSPYNSRFESVAWSASACHDGISWLTIGISGPDNGAVAVLKYNGII 212
H3N2 TPYRTLLMNELGVPF-HLGTKQVCIAWSSSSCHDGKAWLHVCITGDDKNATASFIYNGRL 211
H5N1 SPHRTLMSCPVGEAPSPYNSRFESVAWSASACHDGTSWLTIGISGPDNGAVAVLKYNGII 192
H7N9 SQYRALISWPLSSPPTVYNSRVECIGWSSTSCHDGKSRMSICISGPNNNASAVVWYNRRP 208
B1   NKLRHLISVKLGKIPTVENSIFHMAAWSGSACHDGKEWTYIGVDGPDNNALLKVKYGEAY 210
B2   NKLRHLISVKLGKIPTVENSIFHMAAWSGSACHDGREWTYIGVDGPDSNALLKIKYGEAY 210
     .  * *:   :.      .:     .**.::****     : : * :..*   . *.
H1N1 TDTIKSWRNNILRTQESECACVNGSCFTVMTDGPSDGQASYKIFRIEKGKIVKSVEMNAP 272
H3N2 VDSVVSWSKDILRTQESECICINGTCTVVMTDGSASGKADTKILFIEEGKIVHTSTLSGS 271
H5N1 TDTIKSWRNNILRTQESECACVNGSCFTVMTDGPSSGQASYKIFKMEKGKVVKSVELDAP 252
H7N9 VAEINTWARNILRTQESECVCHNGVCPVVFTDGSATGPADTRIYYFKEGKILKWESLTGT 268
B1   TDTYHSYANKILRTQESACNCIGGNCYLMITDGSASGVSECRFLKIREGRIIKEIFPTGR 270
B2   TDTYHSYAKNILRTQESACNCIGGDCYLMITDGPASGISECRFLKIREGRIIKEIFPTGR 270
     .    :: ..******* * * .* *  ::*** : * :. ::  :.:*::::     .
H1N1 NYHYEECSCY-PDSSEITCVCRDNWHGSNRPWVSFNQN-LEYQIGYICSGIFGDNPRPND 330
H3N2 AQHVEECSCY-PRYPGVRCVCRDNWKGSNRPIVDINIKDHSIVSSYVCSGLVGDTPRKND 330
H5N1 NYHYEECSCY-PDAGEITCVCRDNWHGSNRPWVSFNQN-LEYQIGYICSGVFGDNPRPND 310
H7N9 AKHIEECSCY-GERTGITCTCRDNWQGSNRPVIQIDPVAMTHTSQYICSPVLTDNPRPND 327
B1   VKHTEECTCGFASNETIECACRDNSYTAKRPFVKLNVETDTAEIRLMCTDTYLDTPRPND 330
B2   VKHTEECTCGFASNKTIECACRDNSYTAKRPFVKLNVETDTAEIRLMCTKTYLDTPRPND 330
       * ***:*       : *:****   ::** :.::          :*:    *.** **
                       347
H1N1 KTGS---CGPVSSNGANGVKGF----SFKYGNGVWIGRTKSISSRNGFEMIWDP--NGWT 381
H3N2 SSSSSHCLDPNNEEGGHGVKGW----AFDDGNDVWMGRTINETSRLGYETFKVI--EGWS 384
H5N1 GTGS---CGPVFSNGAYGVKGF----SFKYGNGVWIGRTKSTNSRSGFEMIWDP--NGWT 361
H7N9 PN-IGKCNDPYPGNNNNGVKGF----SYLDGANTWLGRTISTASRSGYEMLKVP--NALT 380
B1   GSITGPCES-NGDKGSGGIKGGFVHQRMESKIGRWYSRTMSKTERMGMGLYVKYDGDPWA 389
B2   GSITGPCES-DGDEGSGGIKGGFVHQRMASKIGRWYSRTMSKTKRMGMGLYVKYDGDPWT 389
      .      .    :.  *:**           . * .** .  .* *         :  :
H1N1 GTDNNFSI-KQDIVGINEWSGYSGSFVQHPELTGLDCIKPCFWVELIRGRPKENT-IWTS 439
H3N2 NPKSKLQTNRQVIVDRGDRSGYSGIFSV----EGKSCINRCFYVELIRGRKEETEVLWTS 440
H5N1 GTDSSFSV-KQDIVAITDWSGYSGSFVQHPELTGLDCIRPCFWVELIRGRPKEST-IWTS 419
H7N9 DDRSKPIQ-GQTIVLNADWSGYSGSFMDY--WAEGDCYRACFYVELIRGRPKEDKVWWTS 437
B1   DSDALAF--SGVMVSMKEPGWYSF----GFEIKDKKCDVPCIGIEMVHDGGKE---TWHS 440
B2   DSEALAL--SGVMVSMEEPGWYSF----GFEIKDKKCDVPCIGIEMVHDGGKT---TWHS 440
     .           :*   : . **            .*   *: :*:::.  :     * *
H1N1 GSSISFCGVNSDTVGWSWPDGAELPFTIDK 469
H3N2 NSIVVFCGTSGTYGTGSWPDGADLNLMPI- 469
H5N1 GSSISFCGVNSDTVSWSWPDGAELPFTIDK 449
H7N9 NSIVSMCSSTEFLGQWNWPDGAKIEYFL-- 465
B1   AATAIYCLMGSGQLLWDTVTGVDMAL---- 466
B2   AATAIYCLMGSGQLLWDTVTGVNMTL---- 466
      :    *         .   *..:
```

Supplementary figure S6. Sequence comparison of neuraminidase of six influenza strains.
H1N1: A/Brisbane/10/2010 (GSAID accession no. EPI745533); H3N2: A/Hong Kong/4801/2014 (GSAID accession no. EPI614413); H5N1: A/turkey/Turkey/1/2005 (GSAID accession no. EPI118777); H7N9: A/Anhui/1/2013 (GSAID accession no. EPI439509); Influenza B1: B/Brisbane/60/2008 (GSAID accession no. EPI2082060); Influenza B2: B/Phuket/3073/2013 (GSAID accession no. EPI1649071). The sequence identity of glycated lysine residues in H1N1 is highlighted in red.

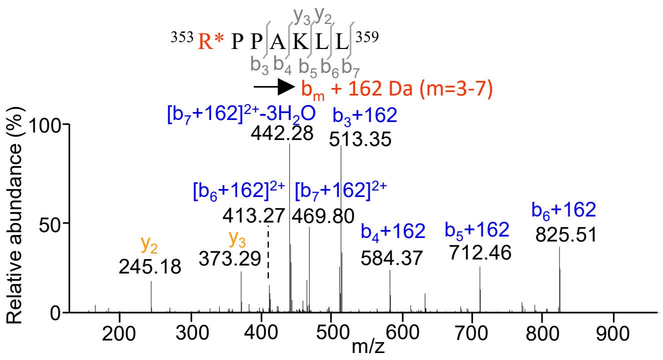

Supplementary figure S7. Hemagglutinin peptide containing hexose-modified arginine. MS/MS spectrum of the doubly charged ion at m/z 478.7920 from a chymotrypsin digest of human Per C6 cell-derived influenza B/Brisbane/60/2008. The mass increase of 162 Da is localized at the N-terminus of hemagglutinin peptide 353-359.

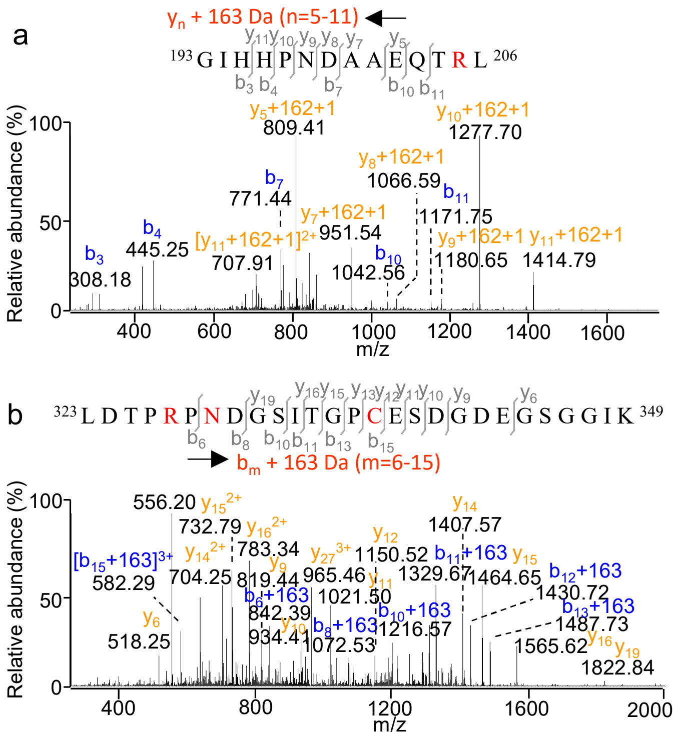

Supplementary figure S8. False-positive identification of the hexose-glycated arginine of hemagglutinin and neuraminidase peptides. (a) MS/MS spectrum of the doubly charged ions at m/z 861.4024 from a chymotrypsin digest of influenza A/turkey/Turkey/1/2005 (H5N1); (b) MS/MS spectrum of the triply charged ion at m/z 965.4288 from a chymotrypsin/ trypsin digest of influenza B/Phuket/3073/2013. The database search identified the peptides at hemagglutinin residues 193-206 and neuraminidase residues 323-349, respectively. The mass increase of 163 Da of the two peptides was tentatively defined as the hexose-glycated arginine (+ 162.0528 Da) and the deamidation of Asn or Gln (+ 0.9840 Da).

Supplementary Table S1. Identification of glycated peptides in influenza A/Brisbane/10/2010 by LC MS/MS analyses and Mascot database search

Protein	Peptide	m/z (Obs.)	Mr (Expt.)	Mr (Calc.)	ppm	Peptide sequence	Site	Enzyme
M1	9-23	875.4789(2+)	1748.9432	1748.9448	-0.89	E.TYVLSIIPSGPLKAE.I	K21	GT
M1	28-47	819.4139(3+)	2455.2198	2455.2192	0.25	R.LESVFAGKNTDLEALMEWLK.T	K35	T
M1	30-40	671.8221(2+)	1341.6297	1341.6300	-0.20	E.SVFAGKNTDLE.A	K35	GT
M1	33-42	597.2988(2+)	1192.5830	1192.5823	0.58	F.AGKNTDLEAL.M	K35	CT
M1	33-45	820.3799(2+)	1638.7453	1638.7447	0.40	F.AGKNTDLEALMEW.L	K35	CT
M1	45-57	429.5079(4+)	1714.0004	1714.0025	-1.27	E.WLKTRPILSPLTK.G	K47	GT
M1	47-55	593.8563(2+)	1185.6970	1185.6964	0.58	L.KTRPILSPL.T	K47	CT
M1	56-62	449.2502(2+)	896.4857	896.4855	0.27	L.TKGILGF.V	K57	CT
M1	95-102	584.3583(2+)	1166.7014	1166.7023	-0.41	D.RAVKLYKK.L	K98	GT
M1	96-100	378.2130(2+)	754.4104	754.4108	-0.49	R.AVKLY.K	K98	CT
M1	106-134	835.1570(4+)	3336.5988	3336.6006	-0.53	R.EITFHGAKEVSLSYSTGALASCMGLIYN R.M	K113	T
M1	106-134	1113.2079(3+)	3336.6018	3336.6006	0.37	R.EITFHGAKEVSLSYSTGALASCMGLIYN R.M	K113	T
M1	107-114	532.7665(2+)	1063.5174	1063.5181	-0.65	E.ITFHGAKE.V	K113	GT
M1	110-119	626.8066(2+)	1251.5986	1251.5983	0.26	F.HGAKEVSLSY.S	K113	CT
M1	179-190	707.8439(2+)	1413.6732	1413.6731	0.06	R.MVLASTTAKAME.Q	K187	GT
M1	179-210	876.9037(4+)	3503.5857	3503.5888	-0.89	R.MVLASTTAKAMEQMAGSSEQAAEAME VANQTR.Q	K187	T
M1	179-210	1168.8705(3+)	3503.5896	3503.5888	0.24	R.MVLASTTAKAMEQMAGSSEQAAEAME VANQTR.Q	K187	T
M1	182-210	1054.4720(3+)	3160.3926	3160.3954	-0.90	L.ASTTAKAMEQMAGSSEQAAEAMEVAN QTR.Q	K187	CT
M1	218-231	766.8750(2+)	1531.7344	1531.7361	-1.14	R.TIGTHPSSSAGLKD.D	K230	GT
M1	218-235	668.3316(3+)	2001.9730	2001.9742	-0.63	R.TIGTHPSSSAGLKDDLLE.N	K230	GT
M1	218-242	712.8607(4+)	2847.4138	2847.4138	0.00	R.TIGTHPSSSAGLKDDLLENLQAYQK.R	K230	T
M1	218-242	950.1448(3+)	2847.4125	2847.4138	-0.44	R.TIGTHPSSSAGLKDDLLENLQAYQK.R	K230	T
M1	218-242	1424.7152(2+)	2847.4159	2847.4138	0.74	R.TIGTHPSSSAGLKDDLLENLQAYQK.R	K230	T
M1	218-243	601.7106(5+)	3003.5168	3003.5149	0.63	R.TIGTHPSSSAGLKDDLLENLQAYQKR.M	K242	T
M1	218-243	751.8862(3+)	3003.5158	3004.4989	0.31	R.TIGTHPSSSAGLKDDLLENLQAYQKR.M	K242	T
HA	63-83	793.4105(3+)	2377.2097	2377.2100	-0.12	R.GVAPLHLGKCNIAGWILGNPE.C	K71	GT
HA	163-167	418.2320 (2+)	834.4484	834.4483	0.19	Y.KNLIW.L	K163	CT
HA	187-194	553.3109(2+)	1104.6073	1104.6067	0.54	K.GKEVLVLW.G	K188	CT
HA	244-260	1134.5234(2+)	2267.0323	2267.0344	-0.91	R.MNYYWTLVEPGDKITFE. A	K256	GT
HA	249-259	691.3583(2+)	1380.7021	1380.7024	-0.22	W.TLVEPGDKITF.E	K256	CT
HA	277-309	1217.5656(3+)	3649.6734	3649.6764	-0.87	R.NAGSGIIISDTPVHDCNTTCQTPKGAINT SLPF.Q 2 deamidated N	K300	CT
HA	277-319	1188.8350(4+)	4751.3088	4751.3059	0.65	R.NAGSGIIISDTPVHDCNTTCQTPKGAINT SLPFQNIHPITIGK.C 2 deamidated N	K300	T
HA	301-322	857.1150(3+)	2568.3233	2568.3258	-0.95	K.GAINTSLPFQNIHPITIGKCPK.Y deamidated N	K319	T
HA	384-395	747.8860(2+)	1493.7564	1493.7573	-0.64	K.STQNAIDKITNK.V	K391	GT
HA	402-412	693.8502(2+)	1385.6859	1386.6861	-0.13	E. KMNTQFTAVGK.E	K402	GT
HA	427-436	685.3300(2+)	1368.6454	1368.6449	0.32	K.KVDDGFLDIW.T	K427	CT
HA	453-463	765.3417(2+)	1528.6689	1528.6682	0.51	L.DYHDSNVKNLY.E	K460	GT
NA	85-103	1094.5073(2+)	2187.0001	2187.0042	-1.86	K.LAGNSSLCPVSGWAIYSKD.N deamidated N	K102	GT
NA	85-107	886.7612(3+)	2657.2617	2657.2643	-0.97	K.LAGNSSLCPVSGWAIYSKDNSIR.I deamidated N	K102	T
NA	143-150	524.2710(2+)	1046.5264	1046.5244	1.98	D.KHSNGTIK.D deamidated N	K143	GT
NA	332-349	930.4031(2+)	1858.7916	1858.7891	1.34	K.TGSCGPVSSNGANGVKGF.S	K347	CT
NA	412-425	950.4468(2+)	1898.8791	1898.8794	-0.16	E.LTGLDCIKPCFWVE.L	K419	GT

The identified glycated peptides are obtained by enzymatic digestion in 25mM ammonium bicarbonate solution at pH 7.6
Enzyme abbreviations: GT: Glu-C digestion flowed by trypsin; CT: chymotrypsin digestion followed by trypsin; T: trypsin digestion only.
The glycated lysine residue in the peptide sequence is highlighted in red
Deamidated modification of peptides is caused by de-N-glycosylation by PNGase F at the NxS/T motif and the liable modification at the NG motif.

Supplementary Table S2. List of the glycated peptides and glycation sites of influenza proteins identified by LC MS/MS, Mascot database search and manual validation

Influenza strain (NIBSIC code)	Matrix 1 (M1)		Hemagglutinin (HA)		Neuraminidase (NA)	
	GISAID Epiflu accession no.	Glycated peptides (glycation sites)	GISAID Epiflu accession no.	Glycated peptides (sites)	GISAID Epiflu accession no.	Glycated peptides (sites)
A/Brisbane/10/2010, H1N1, MDCK cell derived (11/134)	EPI745534 (aa 1-252)	18 peptides (9 sites)	EPI745532 (aa 1-566)	12 peptides (10 sites)	EPI745533 (aa 1-469)	5 peptides (4 sites)
	K21<br>K35<br>K47<br>K57<br>K98<br>K113<br>K187<br>K230<br>K242	9-23 (K21)<br>28-47 (K35)<br>30-40 (K35)<br>33-39 (K35)<br>33-42 (K35)<br>33-45 (K35)<br>47-55 (K47)<br>56-62 (K57)<br>95-102 (K98)<br>96-100 (K98)<br>106-134 (K113)<br>110-119 (K113)<br>179-190 (K187)<br>179-210 (K187)<br>182-210 (K187)<br>218-235 (K230)<br>218-242 (K230)<br>218-243 (K242)	K71<br>K163<br>K188<br>K256<br>K300<br>K319<br>K391<br>K402<br>K427<br>K460	63-83 (K71)<br>163-167 (K163)<br>187-194 (K188)<br>249-259 (K256)<br>244-260 (K256)<br>277-309 (K300)<br>277-319 (K300)<br>301-322 (K319)<br>384-395 (K391)<br>402-412 (K402)<br>427-436 (K427)<br>453-463 (K460)	K102<br>K143<br>K347<br>K419	85-103 (K102)<br>85-107 (K102)<br>143-150 (K143)<br>332-349 (K347)<br>412-425 (K419)
A/Michigan/45/2015 NYMC X-275, H1N1 egg-derived (16/298)	EPI830225 (aa 1-252)	3 peptides (1 site)	EPI830230 (aa 1-566)	ND	EPI830229 (aa 1-469)	ND
	K187	179-210 (K187)<br>179-190 (K187)<br>182-210 (K187)				
A/HongKong/4801/2014, NYMC X-263B, H3N2, egg-derived (16/286)	EPI614409 (aa 1-252)	8 peptides (6 sites)	EPI614414 (aa 1-566)	12 peptides (9 sites)	EPI614413 (aa 1-469)	1 peptide (1 site)
	K21<br>K35<br>K98<br>K113<br>K187<br>K230	9-23 (K21)<br>28-47 (K35)<br>80-99 (K98)<br>106-134 (K113)<br>179-190 (K187)<br>179-210 (K187)<br>182-210 (K187)<br>218-242 (K230)	K99<br>K108<br>K192<br>K205<br>K254<br>K308<br>K326<br>K403<br>K433	99-105 (K99)<br>99-106 (K99)<br>107-125 (K108)<br>177-193 (K192)<br>177-217 (K205)<br>251-271 (K254)<br>295-310 (K308)<br>295-315 (K308)<br>324-331 (K326)<br>400-407 (K403)<br>428-437 (K433)<br>431-442 (K433)	K378	372-387 (K378)
A/turkey/Turkey/1/2005, NIBRG-23, H5N1 (07/112)	EPI118789 (aa 1-252)	2 peptides (2 sites)	EPI118794 (aa 1-568)	13 peptides (7 sites)	EPI118777 (aa 1-449)	1 peptide (1 site)
	K21<br>K187	9-23 (K21)<br>179-190 (K187)	K64<br>K135<br>K250<br>K293<br>K404<br>K429<br>K462	58-68 (K64)<br>455-465 (K462)<br>130-156 (K135)<br>283-320 (K293)<br>398-414 (K404)<br>452-467 (K462)<br>244-253 (K250)<br>244-258 (K250)<br>398-410 (K404)<br>130-138(K135)<br>398-409 (K404)<br>429-438 (K429)<br>455-465 (K462)	K370	368-379 (K370)
A/Anhui/1/2013, NIBRG-268, H7N9, MDCK cell-derived (14/250)	EPI439506 (aa 1-252)	3 peptides (3 sites)	EPI439507 (aa 1-560)	4 peptides (4 sites)	EPI439509 (aa 1-465)	ND
	K57<br>K95<br>K98	56-62 (K57)<br>80-99 (K98)<br>80-100(K95)	K109<br>K175<br>K209<br>K456	91-110 (K109)<br>161-177 (K175)<br>205-218 (K209)<br>434-457 (K456)		

B/Brisbane/60/2008, egg-derived, (13/234)	EPI366462<br>(aa 1-248)	4 peptides<br>(4 sites)	EPI366461<br>EPI2082061<br>(aa 1-585)	3 peptides<br>(3 sites)	EPI366463<br>EPI2082060<br>(aa 1-466)	1 peptide<br>(1 sites)
	K35<br>K187<br>K200<br>K227	28-47 (K35)<br>176-194 (K187)<br>195-213 (K200)<br>221-234 (K227)	K341<br>K412<br>K486	332-344 (K341)<br>410-421 (K412)<br>486-503 (K486)	K343	339-349 (K343)
B/Brisbane/60/2008 NYMC BX-35, Per C6. cell-derived, (14/146)	EPI366462<br>(aa 1-248)	ND	EPI366461<br>EPI2082061<br>(aa 1-585)	2 peptides<br>(2 sites)	EPI366463<br>EPI2082060<br>(aa 1-466)	1 peptide<br>(1 site)
			K331<br>K401	329-341 (K331)<br>401-409 (K401)	K343	339-349 (K343)
B/Phuket/3073/2013, egg derived (16/158)	EPI1649067<br>(aa 1-248)	6 peptides<br>(5 sites)	EPI1649072<br>(aa 1-584)	14 peptides<br>(11 sites)	EPI1649071<br>(aa 1-466)	1 peptide<br>(1 site)
	K35<br>K153<br>K200<br>K227<br>K234	28-47 (K35)<br>144-167 (K153)<br>195-213 (K200)<br>221-234(K227)<br>221-245 (K227 and K234)<br>228-245(K234)	K53<br>K67<br>K131<br>K181<br>K212<br>K216<br>K226<br>K298<br>K330<br>K422<br>K485	33-60(K53)<br>39-60 (K53)<br>66-95 (K67)<br>131-141 (K131)<br>178-197 (K181)<br>182-216 (K212)<br>211-226 (K212, and K216)<br>217-259 (K226)<br>220-238 (K226)<br>296-319 (K298)<br>328-340 (K330)<br>412-426 (K422)<br>485-500 (K485)<br>485-502 (K485)	K107	103-113 (K107)

ND: not detected lysine glycation. The number of glycated peptides is only shown the unique peptides identified by Mascot database search.

Supplementary Table S3. Motif analysis of sequences surrounding the glycation sites of influenza membrane proteins matrix 1, hemagglutinin and neuraminidase

Sequence fragment	Protein	Glycation sites	Polar amino acid-containing
LESVFAGKNTDLEAL	EPI745534	K35	
EITFHGAKEVSLSYS	EPI745534	K113	
VLASTTAKAMEQMAG	EPI745534	K187	S
PSSSAGLKDDLLENL	EPI745534	K230	S
ENLQAYQKRMGVQMQ	EPI745534	K242	
IIPSGPLKAEIAQRL	EPI745534	K21	S
NNMDRAVKLYKKLKR	EPI745534	K98	D K
EALMEWLKTRPILSP	EPI745534	K47	
PILSPLTKGILGFVF	EPI745534	K57	S
VAPLHLGKCNIAGWI	EPI745532	K71	
AGAKSFYKNLIWLVK	EPI745532	K163	K
SYINDKGKEVLVLWG	EPI745532	K188	
TLVEPGDKITFEATG	EPI745532	K256	E
NTTCQTPKGAINTSL	EPI745532	K300	
IHPITIGKCPKYVKS	EPI745532	K319	
STQNAIDKITNKVNS	EPI745532	K391	K
KVNSVIEKMNTQFTA	EPI745532	K402	S
RIENLNKKVDDGFLD	EPI745532	K427	
DYHDSNVKNLYEKVR	EPI745532	K460	D,E
SGWAIYSKDNSIRIG	EPI745533	K102	
QGALLNDKHSNGTIK	EPI745533	K143	
SNGANGVKGFSFKYG	EPI745533	K347	
LTGLDCIKPCFWVEL	EPI745533	K419	
VLASTTAKAMEQMAG	EPI830225	K187	S
IIPSGPLKAEIAQRL	EPI614409	K21	S
LEDVFAGKNTDLEVL	EPI614409	K35	
NNMDKAVKLYRKLKR	EPI614409	K98	D K
EITFHGAKEISLSYS	EPI614409	K113	
VLASTTAKAMEQMAG	EPI614409	K187	S
PSSSAGLKNDLLENL	EPI614409	K230	S
CDGFQNKKWDLFVER	EPI614414	K99	
DLFVERSKAYSSCYP	EPI614414	K108	S
PNNEQFDKLYIWGVH	EPI614414	K192	E
VHHPGTDKDQIFPYA	EPI614414	K205	
SIYWTIVKPGDILLI	EPI614414	K254	
NGSIPNDKPFQNVNR	EPI614414	K308	
GACPRYVKHSTLKLA	EPI614414	K326	
KLNRLIGKTNEKFHQ	EPI614414	K403	K,R
EKYVEDTKIDLWSYN	EPI614414	K433	
RLGYETFKVIEGWSN	EPI614413	K378	
IIPSGPLKAEIAQKL	EPI118789	K21	S
VLASTTAKAMEQMAG	EPI118789	K187	S
LCDLDGVKPLILRDC	EPI118794	K64	
EKIQIIPKSSWSDHE	EPI118794	K135	S
EFFWTILKPNDAINF	EPI118794	K250	
EYGNCNTKCQTPIGA	EPI118794	K293	
KVNSIIDKMNTQFEA	EPI118794	K404	S
RIENLNKKMEDGFLD	EPI118794	K429	
DFHDSNVKNLYDKVR	EPI118794	K462	D
TDSSFSVKQDIVAIT	EPI118777	K370	S
PILSPLTKGILGFVF	EPI439506	K57	S
GDPNNMDKAVKLYKK	EPI439506	K95	
NNMDKAVKLYKKLKR	EPI439506	K98	D K
SDVCYPGKFVNEEAL	EPI439507	K109	E
AAFPQMTKSYKNTRK	EPI439507	K175	
KLYGSGNKLVTVGSS	EPI439507	K209	
LADSEMDKLYERVKR	EPI439507	K456	S R
LHCWFGGKEFDLDSA	EPI366462	K35	
VSAMNTAKTMNGMGK	EPI366462	K187	

GKGEDVQKLAEEELQS	EPI366462	K200		E	
KNGEGIAKDVMEVLK	EPI366462	K227		E	
GNCPIWVKTPLKLAN	EPI366461	K341			K
EAINKITKNLNSLSE	EPI366461	K412	S		
ALERKLKKMLGPSAV	EPI366461	K486			R
PCESNGDKGSGGIKG	EPI366463	K343	S		
YYTGEHAKAIGNCPI	EPI366461	k331			
VAVAADLKSTQEAIN	EPI366461	k401		E	
PCESNGDKGSGGIKG	EPI366463	K343	S		
LHCWFGGKEFDLDSA	EPI1649067	K35			
TLCALCEKQASHSHR	EPI1649067	K153			
GKGEDVQKLAEEELQS	EPI1649067	K200		E	
KNGEGIAKDVMEVLK	EPI1649067	K227		E	
KDVMEVLKQSSMGNS	EPI1649067	K234			
PLTTTPTKSYFANLK	EPI1649072	K53	T		
KGTRTRGKLCPDCLN	EPI1649072	K67		D	R
NLLRGYEKIRLSTQN	EPI1649072	K131	S		R
AVPKDNYKNATNPLT	EPI1649072	K181			K
WGFHSDNKTQMKSLY	EPI1649072	K212			K
SDNKTQMKSLYGDSN	EPI1649072	K216			K
YGDSNPQKFTSSANG	EPI1649072	K226	S		
SGRSKVIKGSLPLIG	EPI1649072	K298	S		
YYTGKHAKAIGNCPI	EPI1649072	K330			
SLSELEVKNLQRLSG	EPI1649072	K422		E	R
ALERKLKKMLGPSAV	EPI1649072	K485			R
PHRFGETKGNSAPLI	EPI1649071	K107			
Subtotal numbers			24	15	16

A total of 85 sequences containing 15 amino acid residues at the N- and C-terminal side of the glycation sites are analyzed.
Polar amino acids of the sequence fragments at ± 4 positions are highlighted in different colours: S/T, pink; D/E, blue; K/R, Green.

Supplementary Table S4. Influenza vaccine materials

Code	Subtype	Influenza vaccine strain	Passage	Composition
13/164	A/H1N1	A /California/7/2009 (H1N1) NYMC X-179A	Eggs	35μg HA/ml, Formalin inactivated, PBS buffer containing 1% (w/v) sucrose
11/134	A/H1N1-like	A/Brisbane/10/2010	MDCK cells	83μg HA/ml. BPL inactivated, PBS buffer containing 1% (w/v) sucrose
16/298	A/H1N1	A/Michigan/45/2015, NYMC X-275	Eggs	38 μg HA/ml, Formalin inactivated, PBS buffer containing 1% (w/v) sucrose
16/286	A/H3N2	A/Hong Kong/4801/2014, NYMC X-263B	Eggs	60 μg HA/ml, Formalin inactivated PBS buffer containing 1% (w/v) sucrose
13/162	A/H3N2-like	A/Texas/50/2012 NMYC X-223A	MDCK cells	72μg HA/ml, BPL inactivated, PBS buffer containing 1% (w/v) sucrose
07/112	A/H5N1	A/turkey/Turkey/1/2005, NIBRG-23	MDCK cells	80μg HA/ml, Formalin inactivated, PBS buffer containing 1% (w/v) sucrose
07/290	A/H5N1	A/Anhui/1/05(H5N1) IBCDC-RG-6	Unkown	99μg HA/ml, BPL inactivated, PBS buffer containing 1% (w/v) sucrose
14/250	A/H7N9	A/Anhui/1/2013, NIBRG-268,	MDCK cells	37μg HA/ml, BPL inactivated, PBS buffer containing 1% (w/v) sucrose
13/234	Influenza B	B/Brisbane/60/2008, NYMC BX-35	Eggs	42μg HA/ml, Formalin inactivated, PBS buffer containing 1% (w/v) sucrose
16/118	Influenza B	B/Brisbane/60/2008, NYMC BX-35	Eggs	44 μg HA/ml, Formalin inactivated, PBS buffer containing 1% (w/v) sucrose
14/146	Influenza B	B/Brisbane/60/2008, NYMC BX-35	Per C6® cells	34μg HA/ml., BPL inactivated, PBS buffer containing 1% (w/v) sucrose
16/158	Influenza B	B/Phuket/3073/2013	Eggs	60 μg HA/ml, Formalin inactivated, PBS buffer containing 1% (w/v) sucrose

The material information of influenza antigens is collected from the NIBSC documents (https://nibsc.org/documents).
Abbreviations: BPL, beta-propiolactone; PBS phosphate-buffered saline solution; HA, haemagglutinin.
